# Graphical Dirichlet Process for Clustering Non-Exchangeable Grouped Data

**Published:** 2024

**Authors:** Arhit Chakrabarti, Yang Ni, Ellen Ruth A. Morris, Michael L. Salinas, Robert S. Chapkin, Bani K. Mallick

**Affiliations:** Department of Statistics, Texas A&M University, College Station, TX 77843-3143, USA; Department of Statistics, CPRIT Single Cell Data Science Core, Texas A&M University, College Station, TX 77843-3143, USA; Department of Nutrition, Program in Integrative Nutrition & Complex Diseases, Current address: Texas A&M Veterinary Medical Diagnostic Laboratory, Texas A&M University College Station, TX 77843-4471, USA; Department of Nutrition, Program in Integrative Nutrition & Complex Diseases, CPRIT Single Cell Data Science Core, Texas A&M University, College Station, TX 77843-2253, USA; Department of Nutrition, Program in Integrative Nutrition & Complex Diseases, CPRIT Single Cell Data Science Core, Texas A&M University, College Station, TX 77843-2253, USA; Department of Statistics, Texas A&M University, College Station, TX 77843-3143, USA

**Keywords:** Bayesian nonparametrics, clustering, directed acyclic graph, family-owned restaurant process, non-exchangeable groups

## Abstract

We consider the problem of clustering grouped data with possibly non-exchangeable groups whose dependencies can be characterized by a known directed acyclic graph. To allow the sharing of clusters among the non-exchangeable groups, we propose a Bayesian nonparametric approach, termed graphical Dirichlet process, that jointly models the dependent group-specific random measures by assuming each random measure to be distributed as a Dirichlet process whose concentration parameter and base probability measure depend on those of its parent groups. The resulting joint stochastic process respects the Markov property of the directed acyclic graph that links the groups. We characterize the graphical Dirichlet process using a novel hypergraph representation as well as the stick-breaking representation, the restaurant-type representation, and the representation as a limit of a finite mixture model. We develop an efficient posterior inference algorithm and illustrate our model with simulations and a real grouped single-cell data set.

## 1 Introduction

This article considers clustering of grouped data where the groups are *non-exchangeable*. We are interested in settings where the data are *partially exchangeable* (de Finetti, 1938), which entails the exchangeability of the observations within each group but not across the groups, (see Kallenberg, 2005 for an extensive bibliography). We consider dependent group–specific random probability measures, thereby allowing the borrowing of information across non-exchangeable groups. We represent the dependencies among groups through a known *directed acyclic graph* (DAG) with nodes denoting groups and directed edges denoting the group dependencies. Such data are abundant in many areas such as genomics. For example, our motivating application is a single-cell RNA-sequencing (scRNA-seq) study that aimed to investigate intestinal stem cell differentiation processes in mice with colorectal cancer. The experiments started from a baseline group where the mice were genetically wild-type, fed with a normal diet, and treated with no cancer therapy (placebo). Then to understand the main effects of genotype, diet, and cancer therapy on colonic crypt and tumor niche cell composition, the experimenters introduced three new groups of mice, each differing from the baseline group by exactly one factor (Apc knock-out, a high-fat diet, or a new cancer treatment AdipoRon). To determine the two-way interaction effects, three additional groups of mice were studied, each of which differed from the baseline group by two factors (e.g., mice with Apc knock-out, a high-fat diet, and no cancer treatment). Lastly, for a three-way interaction, they introduced the eighth group of mice with Apc knock-out, a high-fat diet, and the new treatment AdipoRon. The progression of these experiments from baseline to the study of main effects, two-way interactions, and three-way interactions manifests the non-exchangeability of the experimental groups (e.g., the baseline group is expected to be more similar to the “main effect” groups than the “three-way interaction” group). With this grouped scRNA-seq data set, our goal is to cluster cells based on gene expression at the single-cell level within each experimental group while allowing information to be shared across these non-exchangeable groups with a novel DAG-based Bayesian nonparametric model.

**Figure 1:**
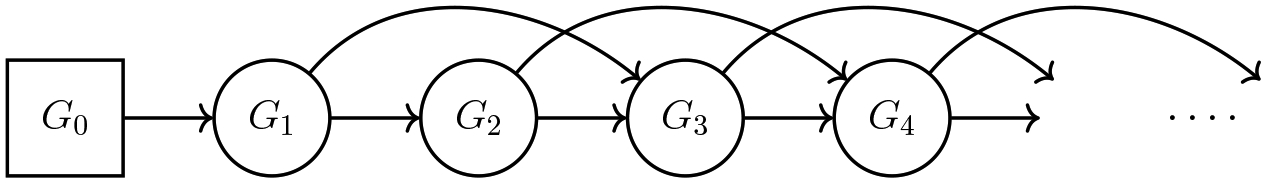
DAG for AR2.

**Figure 2:**
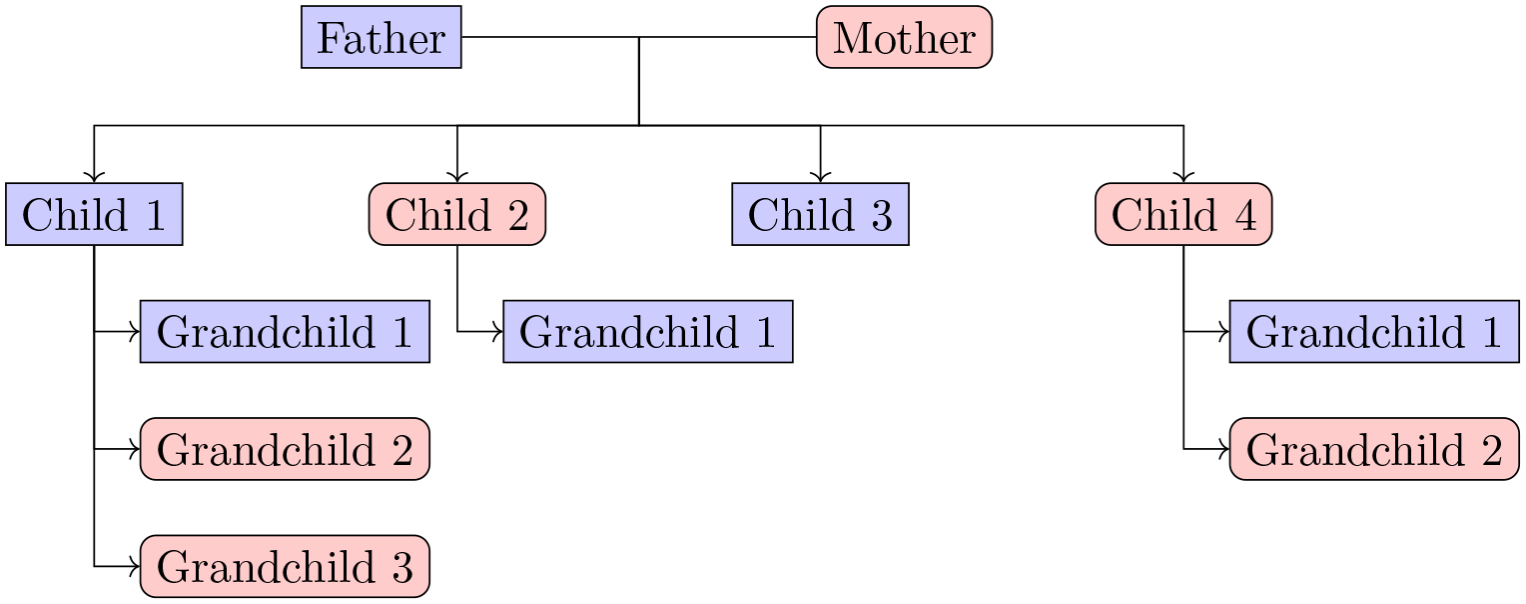
Family tree with three generations. Males are depicted with the color blue and females with red.

Our proposed model extends beyond the specific motivating problem previously discussed. Grouped data can emerge across various disciplines, where the groups exhibit inherent non-exchangeability. Furthermore, the dependencies among the groups can be naturally represented through a known DAG. The first example is time-series data. One might be interested in clustering stocks based on daily prices for each year. Each calendar year is then a group. The groups naturally have time dependence (i.e., one does not expect the clustering of stocks to change dramatically in consecutive years), which may be represented by an autoregressive (AR) model. AR model is one type of DAG model; see Figure 1 for the underlying DAG of AR(2). The second example arises in family tree (Figure 2, another type of DAG). For example, it may be of interest to study the evolution of the gene expressions of a family of three generations to understand how the expression patterns change with the generations. Clustering the gene expression of the family members, where the dependencies between the members (each family member constitutes a group) are naturally explained by the underlying tree, may provide valuable information to the understanding of phenotypic features and/or disease progression (e.g., hemophilia, cancer, etc.).

The *Dirichlet process* (DP, Ferguson, 1973) and its variations (De Blasi et al., 2013; Barrios et al., 2013) have been the backbone of numerous model-based Bayesian nonparametric clustering methods (Hjort et al., 2010; Müller et al., 2015). The DP, $DP\left(\alpha_{0},G_{0}\right)$, is a probability measure on probability measures, where $\alpha_{0}>0$ is the concentration parameter and $G_{0}$ is a base probability measure. There have been extensive studies on DP mixture models (Antoniak, 1974; Escobar and West, 1995; MacEachern and Müller, 1998), which enable clustering without having to fix the number of clusters *a priori*. When there are groups present in the data, naively, one could consider either a separate DP mixture model for each group on one extreme or a single DP mixture model ignoring the groups on the other extreme. However, it is often desirable to identify group-specific clusters while allowing the groups to be linked so that clusters are comparable across groups. Given the goal of clustering the observations within each group, consider a set of random probability measures, $G_{j}$, one for each group j, where each $G_{j}$ is distributed as $DP\left(\alpha_{0j},G_{0j}\right)$ with group-specific concentration parameter $\alpha_{0j}$ and base probability measure $G_{0j}$. Many methods have been proposed to link these group-specific DPs to induce dependencies through the parameter $\alpha_{0j}$ and/or $G_{0j}$ (Cifarelli and Regazzini, 1978; Mallick and Walker, 1997; Kleinman and Ibrahim, 1998; Müller et al., 2004). Perhaps one of the most well-known methods is the hierarchical Dirichlet process (HDP, Teh et al., 2006), which falls in the general framework of dependent DP (MacEachern, 1999, 2000) and assumes each group-specific $G_{j}$ is distributed as $DP\left(\alpha_{0},G_{0}\right)$ where $\alpha_{0}$ is the shared concentration parameter and $G_{0}$ is the shared base probability measure for all groups. They further assume that $G_{0}$ follows another DP, $G_{0}\sim DP(\gamma ,H)$. Since draws from a DP are discrete with probability one (Sethuraman, 1994), the base measure $G_{0}$ is almost surely discrete, which ensures that the group-specific probability measure $G_{j}$ shares the same set of atoms. The corresponding HDP mixture model is thus capable of identifying group-specific clusters while borrowing strength across groups. By construction, HDP mixture model assumes that both the observations within each group and the groups are exchangeable. Recently, several authors have proposed hierarchies of discrete probability measures extending beyond the hierarchical Dirichlet process (Teh, 2006; Thibaux and Jordan, 2007; Zhou, 2016; Tillinghast et al., 2022). See Teh and Jordan, 2010; Foti and Williamson, 2013 for a summary of non-exchangeable priors for Bayesian nonparametric models. Furthermore, Camerlenghi et al., 2019b provides a distribution theory for the entire class of hierarchical processes. A similar approach with a different scope, the nested DP (Rodríguez et al., 2008), assumes $G_{j}$ follows a DP-distributed random probability measure with another DP as the base measure, $G_{j}\sim Q$ and $Q\sim DP\left(\alpha_{0},DP(\gamma ,H)\right)$. The nested structure allows for the clustering of groups but restricts the clusters of observations within each group to be either identical or completely unrelated across groups. Models based on the nested DP have been widely employed in various contexts (Rodriguez and Dunson, 2014; Graziani et al., 2015; Zuanetti et al., 2018). However, similarly to HDP, nested DP also assumes both the observations within each group and the groups to be exchangeable. Moreover, the nested DP is known to suffer from a degeneracy property (Camerlenghi et al., 2019a)–two distributions sharing even one atom in their support are automatically assigned to the same cluster. Several recent works (Beraha et al., 2021; Lijoi et al., 2022; Bi and Ji, 2023) have been proposed to take advantage of the cluster-sharing feature of the HDP and the group-clustering feature of the nested DP. In contrast to methods relying on the HDP or its variants, some other works rely on models with additive structure or common atoms (Camerlenghi et al., 2019a; Chandra et al., 2023; Denti et al., 2023; D’Angelo et al., 2023; D’Angelo and Denti, 2024). Dependent DP has also been extensively used to model random distributions with various other types of dependencies such as spatial and temporal dependencies (Iorio et al., 2004; De Iorio et al., 2009; Dunson and Herring, 2005; Gelfand et al., 2005; Griffin and Steel, 2006; Nieto-Barajas and Contreras-Cristán, 2014; Dahl et al., 2017); see Quintana et al., 2020 for a recent review of different dependent DPs.

**Figure 3:**
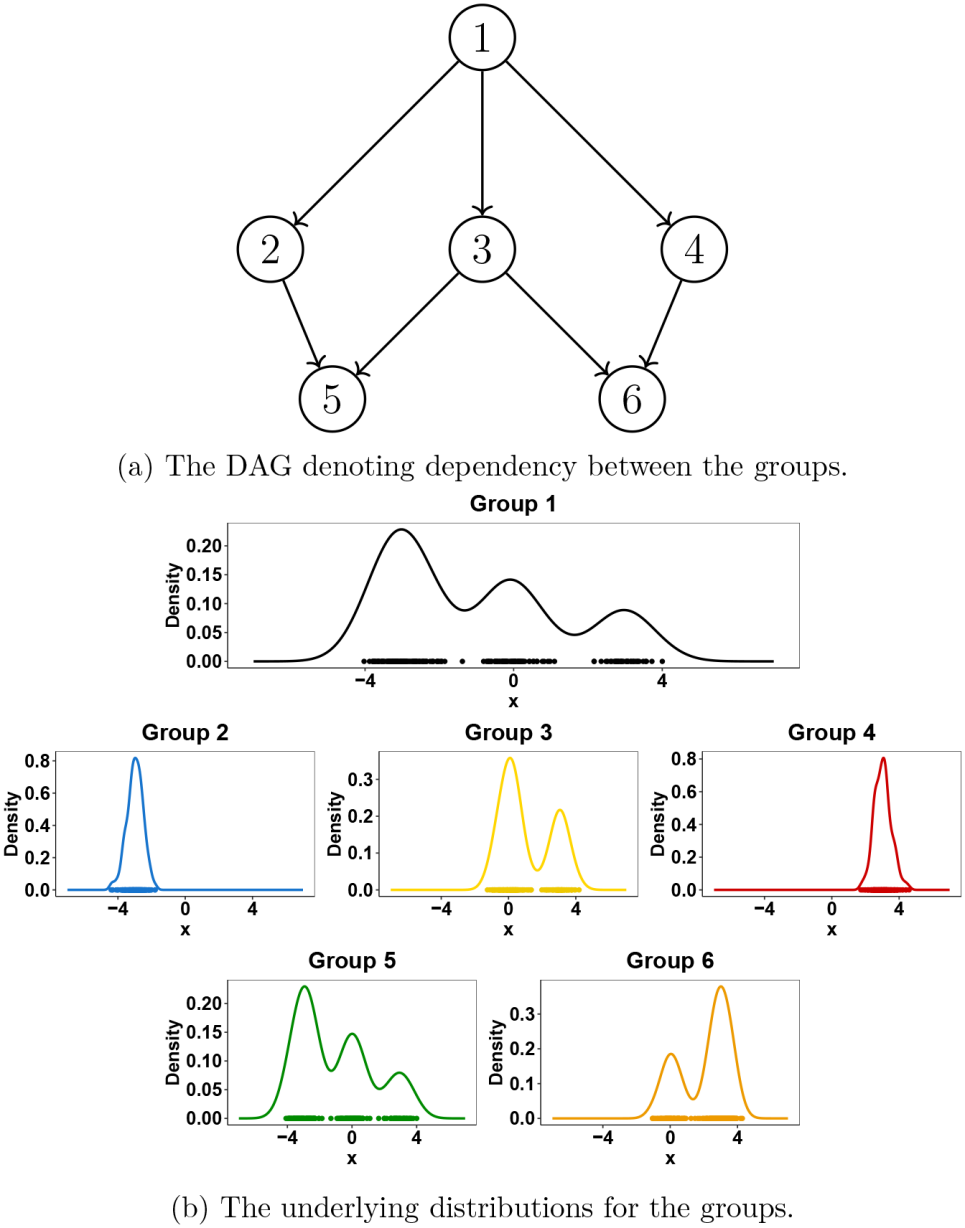
Sharing of features by GDP.

In this paper, we are interested in modeling a set of group-specific random distributions of which the (conditional) dependencies can be characterized by a DAG whose nodes represent the groups. More precisely, we assume that the joint distribution of the set of group-specific random distributions factorizes with respect to a DAG and, therefore, respects its Markov property (i.e., conditional independencies). We call such graph-dependent DP, the *graphical Dirichlet process* (GDP). Using GDP as a mixing distribution, the GDP mixture model gives rise to group-specific clusters, which depend directly on their Markov blanket. As an illustration, for a grouped data with six groups, whose dependencies are shown by the underlying DAG in Figure 3a, the corresponding group-specific distributions are shown in Figure 3b. Clearly, the distributions corresponding to the different groups are similar to their parents. In particular, the distribution of group 6 resembles its parents (group 3 and 4) and is different from the group 5 even though they share a parent (group 3). However, all groups share some or all of the components of the ancestor group (group 1), highlighting the sharing feature of the proposed GDP.

**Figure 4:**
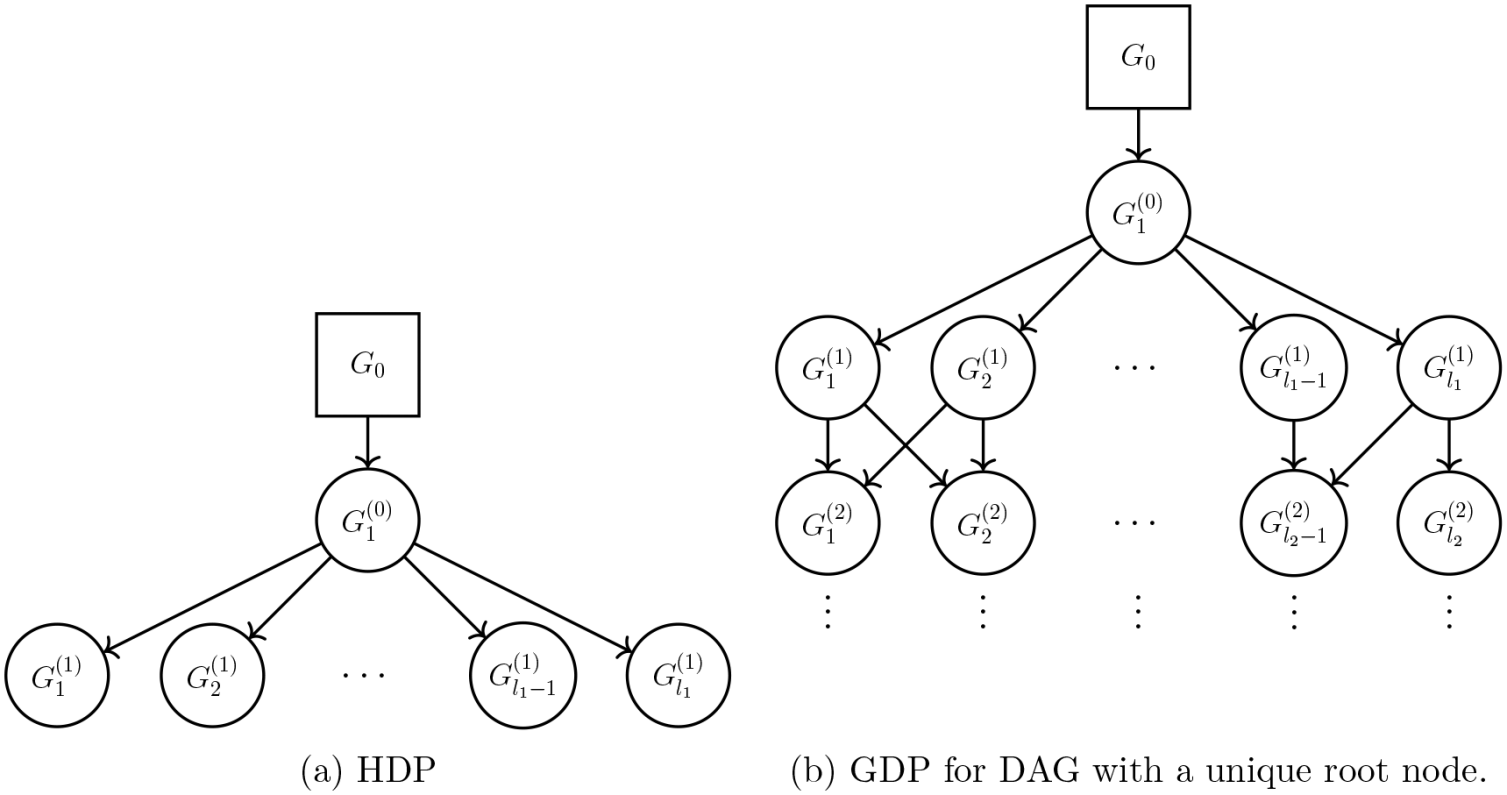
Schematic illustration of HDP and GDP. HDP is a special case of GDP when the DAG is a fork.

The known flexibility of DAG in representing conditional dependencies renders the generality of the proposed GDP for modeling dependent random distributions and group-specific clusters beyond exchangeable groups. The use of DAGs in Bayesian nonparametrics has been considered in recent literature. Dey et al., 2022 proposed a graphical Gaussian process to parsimoniously model multivariate spatial data by incorporating conditional independencies among variables encoded by a DAG. Gu and Dunson, 2023 proposes a pyramid-shaped deep latent variable model for categorical data using a DAG to represent the layer-wise latent conditional dependency structure. These works showcased the usefulness of DAGs through their factorization in Bayesian nonparametric models. We also exploit such factorization in this paper but our model is significantly different from theirs in both approaches and scopes. For example, their graphs link variables whereas ours link groups, and they focus on the modeling of multivariate spatial fields or generative models for categorical data whereas we focus on clustering non-exchangeable grouped data. The proposed GDP is a general model. The well-known HDP is a special case of GDP with a specific type of DAG–a fork, i.e., one parent node and many children nodes (detailed in Section 3.1); see Figure 4a. Several existing works on time-evolving topic models can also be reformulated using a DAG to capture the time-dependency structure (Srebro and Roweis, 2005; Ren et al., 2008; Zhang et al., 2010). Furthermore, the *tree-structured HDP* (Figure 2) considered by Alam et al., 2019 is a special case of our proposed GDP.

In this paper, we will characterize the proposed GDP by a novel *hypergraph* representation, which uses the fact that Dirichlet distribution/process is a normalized gamma distribution/process. We will also provide several other representations analogous to those for the HDP, i.e., a stick-breaking representation, a restaurant-type representation, and a representation as an infinite limit of a finite mixture model. We develop efficient posterior sampling based on the SALTSampler (Director et al., 2017) and a Blocked Gibbs sampler for DP/HDP (Ishwaran and James, 2001; Das et al., 2024). Simulations and the motivating grouped single-cell data are used to demonstrate our method. In summary, our main contribution is three-fold. We propose a general Bayesian nonparametric approach, GDP, to incorporate non-exchangeable group dependencies for clustering. Second, we provide several characterizations of GDP, each providing a different perspective. Furthermore, we develop a Metropolis-within-blocked-Gibbs sampler for posterior inference. Since HDP is a special case of GDP, this also contributes to a new sampler for HDP. The difficulty of sampling the global weights for HDP is mitigated by using the specialized proposal of SALTSampler (Director et al., 2017). Our proposed sampler implementing GDP for the motivational problem is available as an R package, downloadable from the GitHub repository https://github.com/Arhit-Chakrabarti/GDPSamp.

The remainder of the paper is organized as follows. Section 2 provides a brief overview of some preliminaries needed for the remainder of the paper. Section 3 introduces the proposed GDP and the corresponding nonparametric mixture model. We introduce the hyperpriors of our model and also present two lemmas, which are the backbone of our main result in Theorem 3. In Section 4, we present different representations of the proposed GDP. In Section 5, we provide simulations to illustrate our method. Section 6 presents a real data analysis using the proposed method on the motivating single-cell data. The paper concludes with a brief discussion in Section 7.

## 2 Preliminaries

### 2.1 Directed Acyclic Graph

We first provide a brief background on DAG. Let D=(V,E) be a DAG consisting of a set of nodes V={1,2,…,p} and a set of directed edges E⊂V×V that does not contain any directed cycles. We denote a directed edge from the node i to node j by j←i and call i a *parent* of j. A node without parents is called a *root*. For a DAG, there exists at least one root. Let $Y=\left\{Y_{1},\ldots ,Y_{p}\right\}$ be a set of random variables. Every node j∈V represents a random variable $Y_{j}$; later in this paper, $Y_{j}$ will be a random probability measure. In a DAG model, also known as a Bayesian network, the probability distribution 𝒫(Y) is assumed to factorize over $D,𝒫(Y)=\prod_{j=1}^{p}𝒫\left(Y_{j}|Y_{pa(j)}\right)$, where pa(j)={k∈V∣j←k} denotes the collection of parents of node j. This DAG factorization implies that the distribution 𝒫 respects the conditional independence relationships encoded by the graph D via the notion of d-separation (Pearl, 2009); and vice versa. For instance, any node is conditional independent of its non-descendants given its parents, i.e., $Y_{j}⊥Y_{nd(j)}|Y_{pa(j)}$ for any j∈V where ⊥ denotes independence, nd(j)=V∖de(j)∖{j} denotes the non-descendants of node j, and de(j)={k∈V∣k←⋯←j} denotes the descendants of node j. A *Markov blanket* of any node j from V is any subset $V_{1}$ of V such that $Y_{j}⊥Y_{V\setminus V_{1}}|Y_{V_{1}}$. In other words, $V_{1}$ contains all the information in V about the node j. DAG models are convenient tools to parsimoniously specify a multivariate distribution through its conditionals, which is especially useful in this paper for specifying a multivariate distribution of a set of random probability measures.

### 2.2 Infinite Mixture Model

Next, we present a brief overview of infinite mixture models for a single population, the DP mixture model, and for multiple exchangeable populations, the HDP mixture model.

#### 2.2.1 Dirichlet Process Mixture Model

For a single population, let $x_{i}$ denote the ith realization of a random variable X. We consider a mixture model,

(1) $$\theta_{i}|G\overset{iid}{\sim }G,\;x_{i}|\theta_{i}\overset{ind}{\sim }F\left(\theta_{i}\right),$$

where $F\left(\theta_{i}\right)$ denotes the distribution of $x_{i}$ parameterized by $\theta_{i}$. The parameters $\theta_{i}$’s are conditionally independent given the prior distribution G. In a DP mixture model, G is assigned a DP prior, $G\sim DP\left(\alpha_{0},G_{0}\right)$ with concentration $\alpha_{0}$ and base probability measure $G_{0}$.

Sethuraman, 1994 presented the *stick-breaking representation* of the DP based on independent sequences of i.i.d. random variables ${\left(\pi_{k}^{\prime }\right)}_{k=1}^{\infty }$ and ${\left(\phi_{k}\right)}_{k=1}^{\infty }$, which is given by,

(2) $$\pi_{k}^{\prime }|\alpha_{0}\overset{iid}{\sim }\text{Beta}\left(1,\alpha_{0}\right),\;\phi_{k}|G_{0}\overset{iid}{\sim }G_{0},$$

(3) $$\pi_{k}=\pi_{k}^{\prime }\prod_{l=1}^{k-1}\left(1-\pi_{l}^{\prime }\right),\;G=\sum_{k=1}^{\infty }\pi_{k}\delta_{\phi_{k}},$$

where $\delta_{\phi }$ is a point mass at ϕ and $\phi_{k}$’s are called the *atoms* of G. The sequence of random weights $\pi ={\left(\pi_{k}\right)}_{k=1}^{\infty }$ constructed from Eq. (2) and Eq. (3) satisfies $\sum_{k=1}^{\infty }\pi_{k}=1$ with probability one. The random probability measure on the set of integers is denoted by $\pi \sim \text{GEM}\left(\alpha_{0}\right)$ for convenience where GEM stands for Griffiths, Engen and McCloskey (Pitman, 2002). It is clear from Eq. (1) and Eq. (3) that $\theta_{i}$ takes the value $\phi_{k}$ with probability $\pi_{k}$. Let $z_{i}$ be a categorical variable such that $z_{i}=k$ if $\theta_{i}=\phi_{k}$. An equivalent representation of a Dirichlet process mixture is given by,

(4) $$\pi |\alpha_{0}\sim \text{GEM}\left(\alpha_{0}\right),z_{i}|\pi \overset{iid}{\sim }\pi ,\;\phi_{k}|G_{0}\overset{iid}{\sim }G_{0},x_{i}|z_{i},{\left(\phi_{k}\right)}_{k=1}^{\infty }\overset{ind}{\sim }F\left(\phi_{z_{i}}\right).$$

#### 2.2.2 Hierarchical Dirichlet Process Mixture Model

Suppose observations are now organized into multiple exchangeable groups. Let $x_{ji}$ denote the observation i from group j and $\theta_{ji}$ denote the parameter specifying the mixture component associated with the corresponding observation. Let $F\left(\theta_{ji}\right)$ denote the distribution of $x_{ji}$ given $\theta_{ji}$ and $G_{j}$ denote a prior distribution for $\theta_{ji}$. The group-specific mixture model is given by,

(5) $$\theta_{ji}|G_{j}\overset{ind}{\sim }G_{j},\;x_{ji}|\theta_{ji}\overset{ind}{\sim }F\left(\theta_{ji}\right).$$

As with the DP mixture model, when the random measures $G_{j}$’s are assigned an HDP prior,

(6) $$G_{0}|\gamma ,H\sim DP(\gamma ,H),\;G_{j}|\alpha_{0},G_{0}\sim DP\left(\alpha_{0},G_{0}\right),$$

the corresponding mixture model is referred to as the HDP mixture model. The global random probability measure $G_{0}$ is distributed as a DP with concentration parameter γ and base probability measure H. The group-specific random measures $G_{j}$’s are conditionally independent given $G_{0}$ and hence are exchangeable (de Finetti, 1938). They are distributed as DP with the base measure $G_{0}$ and some concentration parameter $\alpha_{0}$. The probability model (5) along with (6) completes the specification of an HDP mixture model. Because DP-distributed $G_{0}$ is almost surely discrete, the atoms of $G_{j}$’s and hence the group-specific clusters are necessarily shared across groups.

## 3 Graphical Dirichlet Process

When groups are non-exchangeable (e.g., due to study design), the joint distribution of $G_{j}$’s specified by (6) may not be appropriate. Our approach to the problem of sharing clusters among non-exchangeable groups is through specifying a general joint distribution of $G_{j}$’s that respect the Markov property of a DAG D that links the groups. We assume that the underlying DAG D is known and we define the appropriate prior on the nodes of the DAG and refer to the resulting stochastic process on the graph as the graphical Dirichlet process (GDP). We show how this prior can be used in the non-exchangeable grouped mixture model setting.

### 3.1 The Proposed GDP

**Figure 5:**
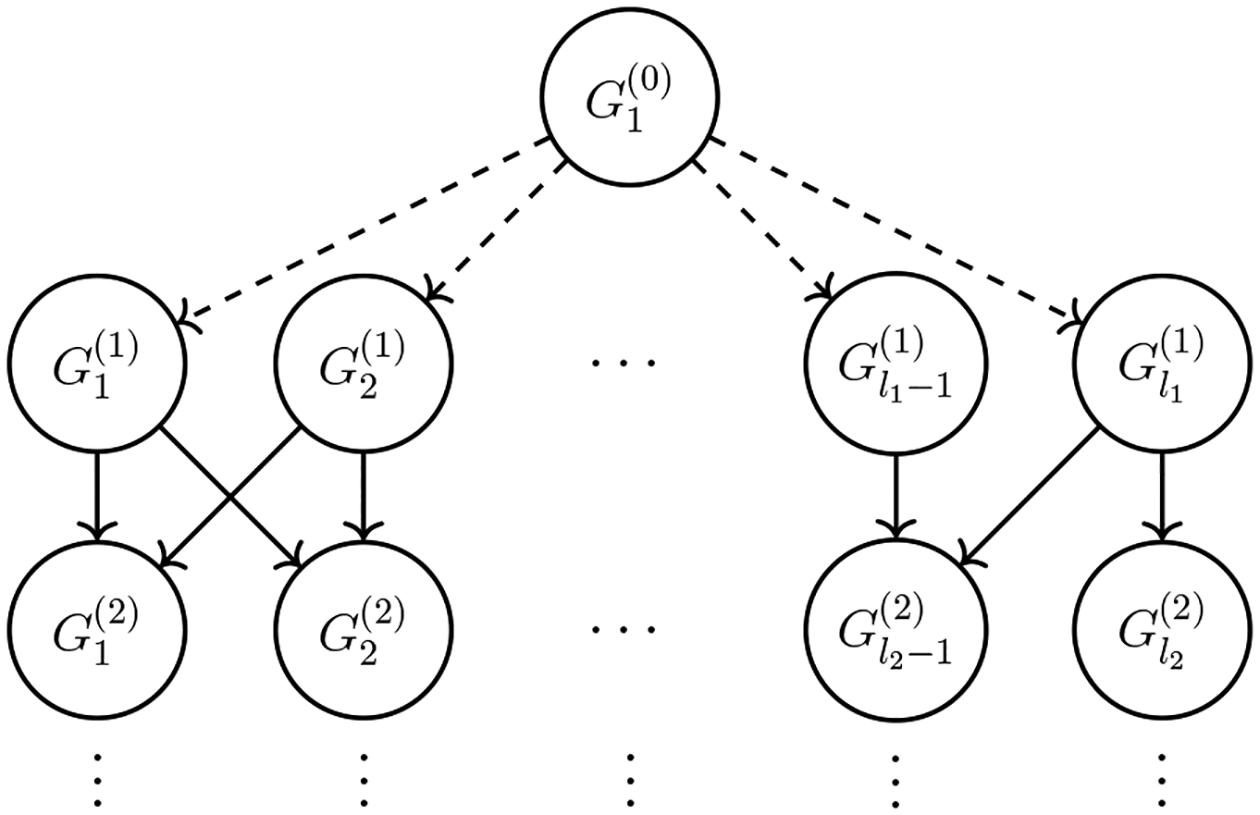
DAG augmented with a hidden root $G_{1}^{\left(0\right)}$, indicated by the dashed arrows. The original root nodes are $G_{1}^{\left(1\right)},\ldots ,G_{l_{1}}^{\left(1\right)}$.

Let the nodes V of DAG D=(V,E) now represent the group-specific random probability measures $G_{j}$’s. The edges E represent the conditional dependence of $G_{j}$’s. Then the joint distribution of the random probability measures follows the DAG factorization $𝒫\left(G_{1},\ldots ,G_{p}|D\right)=\prod_{j=1}^{p}𝒫\left(G_{j}|G_{pa(j)}\right)$, where $G_{pa(j)}$ is the set of random probability measures indexed by the parents pa(j) of node j. For convenience, we assume D has a unique root; see Figure 4b. This assumption does not diminish the generality of our approach as a DAG with multiple roots can always be converted, without losing any conditional dependencies, to a DAG with a unique root by simply augmenting the DAG with a hidden common parent of the roots; that hidden common parent becomes the unique root of the new DAG (Figure 5). The augmentation only changes the Markov blanket of the original root nodes. Specifically, the Markov blanket of any original root node is simply augmented with the hidden parent node. As the Markov blanket of any other node remains unchanged, the distributions of all other nodes remain the same, and hence this augmentation does not alter the conditional dependencies of the original DAG.

Let us introduce a few terms before describing the proposed GDP. We denote the root node, which may be hidden, as the *layer 0* of DAG D. The child nodes of the root node are termed as the *layer-1* nodes, and we assume that there are $l_{1}$ of them. Similarly, we assume that there are a total of $l_{2}$ child nodes from the layer-1 nodes, which we refer to as the layer-2 nodes. We assume that there are K layers in the given DAG D and at any layer k, there are $l_{k}$ nodes. The total number of non-root nodes is $\sum_{k=1}^{K}l_{k}=p$. We define the concentration parameters and random measures of node j in the layer k of DAG D as $\alpha_{j}^{\left(k\right)}$ and $G_{j}^{\left(k\right)},j=1,\ldots ,l_{k}$. We denote by $an^{\left(k,l\right)}(j)$ the collection of generation-l ancestors of node j in layer k of the DAG. For example, $an^{\left(k,1\right)}(j)$ denotes the parents (generation-1 ancestors) of the node j in layer k, and $an^{\left(k,2\right)}(j)$ denotes the collection of the parents of the nodes in $an^{\left(k,1\right)}(j)$ or in other words, $an^{\left(k,2\right)}(j)$ denotes the collection of “grand-parents” (generation-2 ancestors) of node j in layer k of the DAG.

We define GDP recursively from layer 0, the root node,

(7) $$G_{1}^{\left(0\right)}|\alpha_{1}^{\left(0\right)},G_{0}\sim DP\left(\alpha_{1}^{\left(0\right)},G_{0}\right),$$

where $G_{0}$ is a fixed base probability measure. Then the distribution of the random probability measure of node j in layer k of DAG D conditional on the concentration parameters and random probability measures of its parent nodes is given by,

(8) $$G_{j}^{\left(k\right)}|\alpha_{j}^{\left(k\right)},\left\{G_{l}^{\left(k-1\right)}:l\in an^{\left(k,1\right)}\left(j\right)\right\}\sim DP\left(\alpha_{j}^{\left(k\right)},\sum_{l\in an^{\left(k,1\right)}\left(j\right)}\pi_{jl}^{\left(k\right)}G_{l}^{\left(k-1\right)}\right),$$

for $j=1,2,\ldots ,l_{k}$. In other words, node j in layer k of the DAG is distributed according to a DP with its own concentration parameter $\alpha_{j}^{\left(k\right)}$ and its base distribution being a weighted average of the random probability measures of its parents in layer k-1 of the DAG, $\left\{G_{l}^{\left(k-1\right)}:l\in an^{\left(k,1\right)}(j)\right\}$, where the weights are given by $\left\{\pi_{jl}^{\left(k\right)}:l\in an^{\left(k,1\right)}(j)\right\}$, which have a unit sum $\sum_{l\in an^{\left(k,1\right)}(j)}\pi_{jl}^{\left(k\right)}=1$. Moreover, from the Markov properties of DAG $D,G_{j_{1}}^{\left(k\right)}$ and $G_{j_{2}}^{\left(k\right)}$ are conditionally independent given their parents, $\left\{G_{l}^{\left(k-1\right)}:l\in an^{\left(k,1\right)}\left(j_{1}\right)\right\}$ and/or $\left\{G_{l}^{\left(k-1\right)}:l\in an^{\left(k,1\right)}\left(j_{2}\right)\right\}$, and $G_{j}^{\left(k\right)}$ is conditionally independent of all other random probability measures given its Markov blanket.

We remark that HDP is a special case of the proposed GDP with a specific DAG, fork-DAG (Figure 4a). Using the notations introduced, a fork-DAG is a DAG with a unique root node and only one layer of $l_{1}$ child nodes. With this specific DAG, the GDP is given by

$$G_{1}^{\left(0\right)}|\alpha_{1}^{\left(0\right)},G_{0}\sim DP\left(\alpha_{1}^{\left(0\right)},G_{0}\right),\;G_{j}^{\left(1\right)}|\alpha_{j}^{\left(1\right)},G_{1}^{\left(0\right)}\sim DP\left(\alpha_{j}^{\left(1\right)},G_{1}^{\left(0\right)}\right),j=1,2,\ldots ,l_{1},$$

which is clearly an HDP.

### 3.2 GDP Mixture Model

To cluster observations that are organized into possibly non-exchangeable groups, we use the proposed GDP in Section 3.1 as a mixing distribution of a mixture model. Letting j index the groups and i index the observations within each group, we assume that the observations $x_{j1},x_{j2},\ldots ,x_{jn_{j}}$ are exchangeable within each group j but the groups may not be exchangeable. We assume that each observation within a group is drawn independently from the mixture model (5) and $G_{j}$’s follow the GDP (7) and (8).

### 3.3 Hyperpriors

We assign a Dirichlet prior on the weights $\left\{\pi_{jl}^{\left(k\right)}:l\in an^{\left(k,1\right)}(j)\right\}$ in (8),

(9) $$\left\{\pi_{jl}^{\left(k\right)}:l\in an^{\left(k,1\right)}(j)\right\}\sim Dir\left(\left\{\alpha_{l}^{\left(k-1\right)}:l\in an^{\left(k,1\right)}\left(j\right)\right\}\right),$$

where the parameters $\left\{\alpha_{l}^{\left(k-1\right)}:l\in an^{\left(k,1\right)}(j)\right\}$ correspond to the concentration parameters of the parents (generation-1 ancestors) of node j. Since the concentration parameter of a DP relates to its precision (inverse-variance), assuming a Dirichlet prior for the mixture weights of any node with Dirichlet parameters proportional to the precisions of the parent nodes is a natural choice. This gives more “weightage” to a parent node with a higher precision as opposed to a parent node with a lower precision.

The other distributional consideration that significantly simplifies the distribution of the random measure of any particular node is by considering a gamma-DAG distribution on the concentration parameters $\alpha_{j}^{\left(k\right)}$’s, which, like the distribution of $G_{j}^{\left(k\right)}$’s, also respects the same Markov property of DAG D. Specifically, we assume that

(10) $$\alpha_{1}^{\left(0\right)}|\alpha_{0}\sim \text{Gamma}\left(\alpha_{0},1\right),\;\alpha_{j}^{\left(k\right)}|\left\{\alpha_{l}^{\left(k-1\right)}:l\in {an}^{\left(k,1\right)}\left(j\right)\right\}\sim \text{Gamma}\left(\sum_{l\in an^{\left(k,1\right)}\left(j\right)}\alpha_{l}^{\left(k-1\right)},1\right),j=1,2,\ldots ,l_{k}.$$

In other words, the concentration parameter of the root node follows a gamma distribution with a fixed shape $\alpha_{0}$ and a unit rate. The concentration parameter at any level of the DAG follows a conditionally gamma distribution with the shape parameter equal to the sum of the shape parameters of its parents. Such a choice of Gamma hyperprior on the concentration parameters of bottom level DPs of HDP have been considered in Williamson et al., 2013. We extend such a construction for the more general framework of our proposed GDP. In the next section, we will see how our choice of hyperpriors and hyperparameters leads to several compact representations of the proposed GDP, which requires two lemmas. The first lemma is Lemma 3.1 from Sethuraman, 1994, which we state here.

**Lemma 1** (Sethuraman, 1994) *Let* $\alpha_{1}=\left(\alpha_{11},\alpha_{12},\ldots ,\alpha_{1k}\right)$ *and* $\alpha_{2}=\left(\alpha_{21},\alpha_{22},\ldots ,\alpha_{2k}\right)$ *be* k-*dimensional vectors with* $\alpha_{ij}>0\forall j=1,2,\ldots ,k,i=1,2$. *Let* $X_{1}$ *and* $X_{2}$ *be independent* k-*dimensional random vectors distributed as Dirichlet distribution with parameters* $\alpha_{1}$ *and* $\alpha_{2}$, *respectively. Let* $\alpha_{1.}=\sum_{j=1}^{k}\alpha_{1j}$ *and* $\alpha_{2}=\sum_{j=1}^{k}\alpha_{2j}$. *Let* π *be independent of* $X_{1}$ *and* $X_{2}$ *and have a beta distribution Beta* $\left(\alpha_{1},\alpha_{2}.\right)$. *Then the distribution of* $\pi X_{1}+(1-\pi )X_{2}$ *is the Dirichlet distribution with parameter* $\alpha_{1}+\alpha_{2}$.

The proof is provided in Section B of the Appendix for completeness. The next lemma is an immediate extension of Theorem 1 for more than two independent Dirichlet distributed random vectors. As the Dirichlet distribution is a multivariate analog of the beta distribution, by considering a Dirichlet distribution on the weights, we arrive at a similar result. This lemma is a finite-dimensional version of Theorem 1 of Williamson et al., 2013, which essentially states that a finite Dirichlet mixture of DPs is, in turn, a DP with its concentration parameter being the sum of the concentration parameters of the component DPs, and the base measure being a weighted mixture of the corresponding mixing base measures.

**Lemma 2** *Let* $\alpha_{1},\alpha_{2},\ldots ,\alpha_{L}$ *be* k-*dimensional vectors where* $\alpha_{i}=\left(\alpha_{i1},\ldots ,\alpha_{ik}\right)$ *with* $\alpha_{ij}>0\forall j=1,2,\ldots ,k,i=1,2,\ldots ,L.$ *Let* $X_{1},X_{2},\ldots ,X_{L}$ *be independent* k-*dimensional random vectors distributed as Dirichlet distribution with parameters* $\alpha_{1},\alpha_{2},\ldots ,\alpha_{L}$, *respectively. Let* $\alpha_{i}=\sum_{j=1}^{k}\alpha_{ij},i=1,2,\ldots ,L$. *Let* $\pi =\left(\pi_{1},\pi_{2},\ldots ,\pi_{L}\right)$ *be independent of* $X_{1},X_{2},\ldots ,X_{L}$ *and have a Dirichlet distribution Dir* $\left(\alpha_{1},\alpha_{2},\ldots ,\alpha_{L}\right.$.). *Then the distribution of* $\sum_{i=1}^{L}\pi_{i}X_{i}$ *is the Dirichlet distribution with parameter* $\sum_{i=1}^{L}\alpha_{i}$.

The proof is provided in Section B of the Appendix, which uses the fact that Dirichlet distribution is normalized gamma distribution. This lemma will be used to prove the hypergraph representation of GDP in the next section.

## 4 Representations of the Graphical Dirichlet Process

In this section, we characterize the proposed GDP through (i) the hypergraph representation, (ii) the stick-breaking representation, (iii) the restaurant-type process representation, and (iv) the limit of finite mixture representation.

### 4.1 The Hypergraph Representation

The GDP, along with the hyperpriors on the concentration parameters and mixture weights, can be represented hierarchically as,

(11) $$\alpha_{1}^{\left(0\right)}|\alpha_{0}\sim \text{Gamma}\left(\alpha_{0},1\right),\;G_{1}^{\left(0\right)}|\alpha_{1}^{\left(0\right)},G_{0}\sim DP\left(\alpha_{1}^{\left(0\right)},G_{0}\right),\;\alpha_{j}^{\left(k\right)}|\left\{\alpha_{l}^{\left(k-1\right)}:l\in an^{\left(k,1\right)}\left(j\right)\right\}\sim \text{Gamma}\left(\underset{l\in an^{\left(k,1\right)}\left(j\right)}{\sum }\alpha_{l}^{\left(k-1\right)},1\right),\;\left\{\pi_{jl}^{\left(k\right)}:l\in an^{\left(k,1\right)}\left(j\right)\right\}|\left\{\alpha_{l}^{\left(k-1\right)}:l\in an^{\left(k,1\right)}\left(j\right)\right\}\sim Dir\left(\left\{\alpha_{l}^{\left(k-1\right)}:l\in an^{\left(k,1\right)}\left(j\right)\right\}\right),\;G_{j}^{\left(k\right)}|\alpha_{j}^{\left(k\right)},\left\{G_{l}^{\left(k-1\right)}:l\in an^{\left(k,1\right)}\left(j\right)\right\}\sim DP\left(\alpha_{j}^{\left(k\right)},\underset{l\in an^{\left(k,1\right)}\left(j\right)}{\sum }\pi_{jl}^{\left(k\right)}G_{l}^{\left(k-1\right)}\right),$$

for $j=1,2,\ldots ,l_{k}$ and k=1,…,K.

The hyperparameters of the GDP consist of the base probability measure $G_{0}$ and the concentration parameter $\alpha_{0}$. The probability measure $G_{1}^{\left(0\right)}$ of the root node varies around the base measure $G_{0}$ with the amount of variability governed by $\alpha_{1}^{\left(0\right)}$, which in turn is governed by the hyperparameter $\alpha_{0}$. We now present a novel hypergraph representation of GDP, which simplifies the graph-based distribution. The representation follows from the gamma-DAG distribution on the concentration parameters and standard properties of Dirichlet distribution.

**Theorem 3 (Hypergraph Representation)** *Consider a DAG D that has K layers and* $l_{k}$ *distinct nodes in layer* k *for* k=1,…,K. *Under model* (11), *the distribution of the random measure* $G_{j}^{\left(k\right)}$ *of node* j *in layer* k *of DAG D can be equivalently represented as*,

$$G_{j}^{\left(k\right)}|\alpha_{j}^{\left(k\right)},H_{j}^{\left(k,k\right)}\sim DP\left(\alpha_{j}^{\left(k\right)},H_{j}^{\left(k,k\right)}\right),\;H_{j}^{\left(k,k\right)}|\left\{\alpha_{l}^{\left(k-1\right)}:l\in an^{\left(k,1\right)}(j)\right\},H_{j}^{\left(k,k-1\right)}\sim DP\left(\sum_{l\in an^{\left(k,1\right)}(j)}\alpha_{l}^{\left(k-1\right)},H_{j}^{\left(k,k-1\right)}\right),\;H_{j}^{\left(k,k-1\right)}|\left\{\alpha_{l}^{\left(k-2\right)}:l\in an^{\left(k,2\right)}(j)\right\},H_{j}^{\left(k,k-2\right)}\sim DP\left(\sum_{l\in an^{\left(k,2\right)}(j)}\alpha_{l}^{\left(k-2\right)},H_{j}^{\left(k,k-2\right)}\right),\;\vdots \;H_{j}^{\left(k,2\right)}|\left\{\alpha_{l}^{\left(1\right)}:l\in an^{\left(k,k-1\right)}\left(j\right)\right\},G_{1}^{\left(0\right)}\sim DP\left(\sum_{l\in an^{\left(k,k-1\right)}\left(j\right)}\alpha_{l}^{\left(1\right)},G_{1}^{\left(0\right)}\right).$$

**Figure 6:**
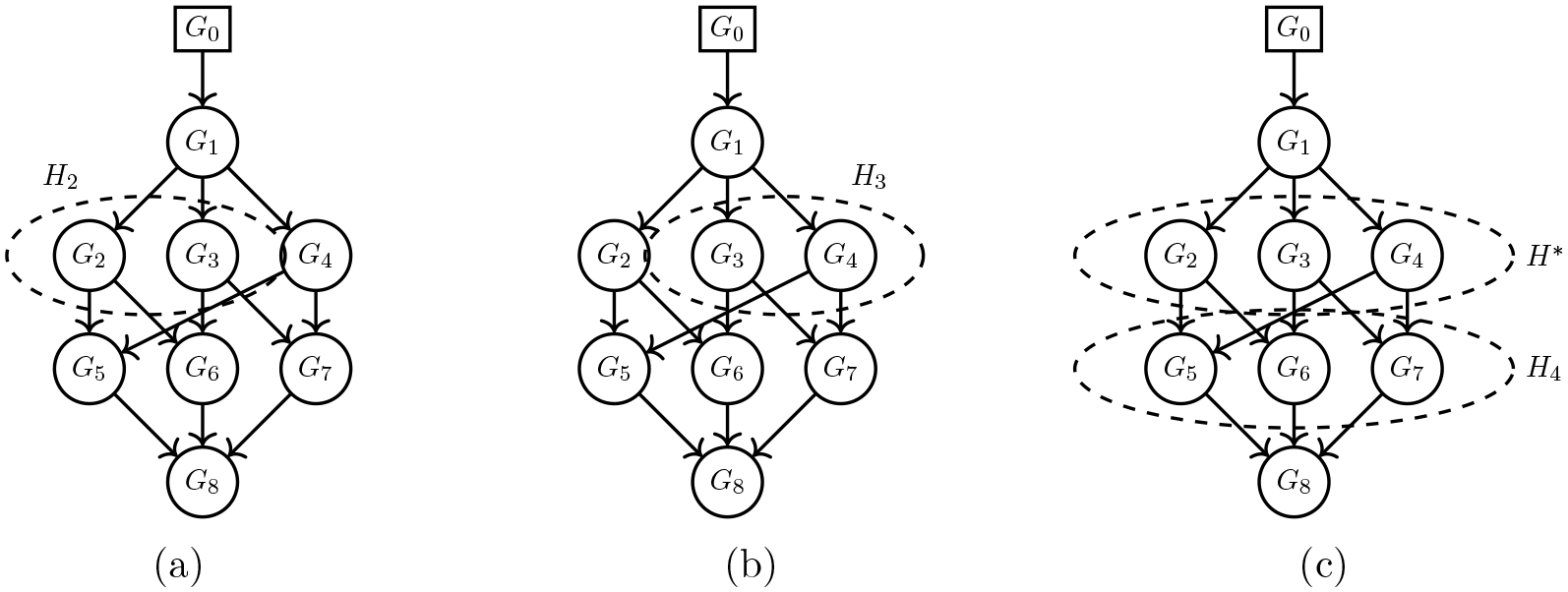
Illustration of hypernodes (represented by dashed ovals) of the DAG for our motivational problem. (a) Hypernode $H_{2}$ consists of the generation-1 ancestors (i.e., $G_{2}$ and $G_{3}$) of node $G_{6}$. (b) Hypernode $H_{3}$ consists of the generation-1 ancestors (i.e., $G_{3}$ and $G_{4}$) of node $G_{7}$. (c) Hypernode $H_{4}$ consists of the generation-1 ancestors (i.e., $G_{5},G_{6}$, and $G_{7}$) of node $G_{8}$. Hypernode $H^{*}$ consists of the generation-2 ancestors (i.e., $G_{2},G_{3}$, and $G_{4}$) of node $G_{8}$.

The proof is provided in the Appendix A. In words, Theorem 3 essentially states the following. The distribution of $G_{j}^{\left(k\right)}$ is a DP with a hidden base measure $H_{j}^{\left(k,k\right)}$ and the concentration parameter $\alpha_{j}^{\left(k\right)}$. The hidden base measure $H_{j}^{\left(k,k\right)}$, in turn, is again a DP with base measure $H_{j}^{\left(k,k-1\right)}$ and concentration parameter being the sum of the concentration parameters of the generation-1 ancestors of $G_{j}^{\left(k\right)}$. Recursively, the hidden base measure $H_{j}^{\left(k,k-1\right)}$ is a DP with base measure $H_{j}^{\left(k,k-2\right)}$ and the concentration parameter being the sum of the concentration parameters of the generation-2 ancestors. This distributional pattern continues in a hierarchical fashion. Through k-1 hidden base measures, any node in layer k can be seen to depend on the root node $G_{1}^{\left(0\right)}$ through its ancestral relationships. We call the representation of GDP in Theorem 3 as the hypergraph representation because one can view $H_{j}^{\left(k,k-a\right)}$ for a=0,…,k-2 as a hypernode that contains all the sufficient information from generation-(a+1) ancestors of $G_{j}^{\left(k\right)}$. We provide in Figure 6 an illustrative example of the hypergraph representation showing how the hypernodes contain all the ancestral information. From Figure 6a, we can see that the distribution of $G_{6}$ depends on the distribution of its parents, $G_{2}$ and $G_{3}$. We refer to $H_{2}$, consisting of $\left\{G_{2},G_{3}\right\}$, as a hypernode. Hypernode $H_{2}$ contains all the information about the parents of $G_{6}$. Loosely speaking, the information of the root node $G_{1}$ (e.g., its atoms) is passed to $G_{6}$ through $H_{2}$. Similarly, $H_{3}$, being the hypernode of $\left\{G_{3},G_{4}\right\}$, contains all the information about $G_{7}$ from its parent nodes allowing the flow of information from the root node (see Figure 6b). For node $G_{8}$, we have two levels of hypernodes–$H_{4}$ denotes the first layer and consists of the parents of $G_{8}$, and $H^{*}$ denotes the second layer and consists of generation-2 ancestors of $G_{8}$. Thus, hypernodes $H_{4}$ and $H^{*}$ carry all the information from the root node $G_{1}$ to $G_{8}$ as illustrated in Figure 6c.

We will exploit this representation to derive the stick-breaking representation and the limit of finite mixture representation of the proposed GDP in the next subsections.

### 4.2 The Stick-Breaking Representation

Given that the random measure $G_{1}^{\left(0\right)}$ of the root node is distributed as a DP, it can be expressed using a stick-breaking representation,

(12) $$G_{1}^{\left(0\right)}=\sum_{l=1}^{\infty }\beta_{1l}^{\left(0\right)}\delta_{\phi_{l}},$$

where $\phi_{l}\overset{iid}{\sim }G_{0}$ and $\beta_{1}^{\left(0\right)}={\left(\beta_{1l}^{\left(0\right)}\right)}_{l=1}^{\infty }\sim \text{GEM}\left(\alpha_{1}^{\left(0\right)}\right)$ are mutually independent. We interpret $\beta_{1}^{\left(0\right)}$ as a probability measure on the positive integers. Since $G_{1}^{\left(0\right)}$ has support at the atoms $\phi ={\left(\phi_{l}\right)}_{l=1}^{\infty }$, each $G_{j}^{\left(k\right)}$ necessarily has support at these atoms as well and hence can be expressed as,

(13) $$G_{j}^{\left(k\right)}=\sum_{l=1}^{\infty }\beta_{jl}^{\left(k\right)}\delta_{\phi_{l}}.$$

As with Theorem 3, the stick-breaking weights depend hierarchically on a set of hidden weights. Letting $\beta_{j}^{\left(k\right)}={\left(\beta_{jl}^{\left(k\right)}\right)}_{l=1}^{\infty }$ be the stick-breaking weights for node j in layer k of DAG D and letting $\nu_{j}^{\left(k,m\right)}={\left(\nu_{jl}^{\left(k,m\right)}\right)}_{l=1}^{\infty },m=2,\ldots ,k$ be their hidden weights, we have the following corollary.

**Corollary 4 (Stick-Breaking Representation)** Consider a DAG D that has K layers and $l_{k}$ *distinct nodes in layer* k *for* k=1,…,K. *The stick-breaking weights* $\beta_{j}^{\left(k\right)}$ *of node* j *at layer* k *of DAG* D *can be represented as*

$$\beta_{j}^{\left(k\right)}|\alpha_{j}^{\left(k\right)},\nu_{j}^{\left(k,k\right)}\sim DP_{Z^{+}}\left(\alpha_{j}^{\left(k\right)},\nu_{j}^{\left(k,k\right)}\right),\;\nu_{j}^{\left(k,k\right)}|\left\{\alpha_{l}^{\left(k-1\right)}:l\in an^{\left(k,1\right)}(j)\right\},\nu_{j}^{\left(k,k-1\right)}\sim DP_{Z^{+}}\left(\sum_{l\in an^{\left(k,1\right)}(j)}\alpha_{l}^{\left(k-1\right)},\nu_{j}^{\left(k,k-1\right)}\right),\;\nu_{j}^{\left(k,k-1\right)}|\left\{\alpha_{l}^{\left(k-2\right)}:l\in an^{\left(k,2\right)}(j)\right\},\nu_{j}^{\left(k,k-2\right)}\sim DP_{Z^{+}}\left(\sum_{l\in an^{\left(k,2\right)}(j)}\alpha_{l}^{\left(k-2\right)},\nu_{j}^{\left(k,k-2\right)}\right),\;\vdots \;\nu_{j}^{\left(k,2\right)}|\left\{\alpha_{l}^{\left(1\right)}:l\in an^{\left(k,k-1\right)}(j)\right\},\beta_{1}^{\left(0\right)}\sim DP_{Z^{+}}\left(\sum_{l\in an^{\left(k,k-1\right)}(j)}\alpha_{l}^{\left(1\right)},\beta_{1}^{\left(0\right)}\right).$$

*where* $DP_{Z^{+}}(a,\eta )$ *denotes the random probability measure on the positive integers distributed as a Dirichlet process with the concentration parameter* a>0 *and base measure on the positive integers*, η.

The proof of this corollary directly follows from the hypergraph representation of Theorem 3 and is hence omitted. We call this representation the *stick-breaking representation* where $\nu_{j}^{\left(k,k\right)}$ is interpreted as a hidden probability measure on the set of positive integers corresponding to the first hidden layer. Each hidden layer of stick-breaking weights depend hierarchically on its previous hidden layer, denoted by $\nu_{j}^{\left(k,k-1\right)},\nu_{j}^{\left(k,k-2\right)}$, and so on, and finally on the weights $\beta_{1}^{\left(0\right)}$ of the root node.

### 4.3 The Family-Owned Restaurant Process Representation

DP and HDP have the well-known Chinese restaurant process and franchise representations. Here, we provide a culinary analog for the proposed GDP. We refer to this process as the *family-owned restaurant process* as it is customary to use familial relationships to describe the relationships between nodes in a DAG. The metaphor is as follows. An original restaurant is opened by the ancestor of a family (the root node), which serves some dishes from a global menu containing an infinite number of dishes. The descendants of the ancestor open their own respective restaurants, which serve some of the dishes already being served in the restaurants owned by their parents and possibly some new dishes from the global menu. At each table of the original restaurant, one dish is ordered from the menu by the first customer occupying the table, and the dish is shared by all the other customers who sit at that table. Any subsequent customer may either join an occupied table and share the dish being served at that table or open a new table with a new dish from the menu. In restaurants other than the original restaurant, however, the first customer might choose to select a dish being served at one of the tables of its parent restaurant or order a new dish from the menu. Since the hypergraph representation of GDP involves hypernodes with hidden probability measures, we introduce a notation for the number of tables serving a dish in any restaurant and demarcate them with the notation for the number of tables serving the dish in the hypernodes, which we refer to as hyper-restaurants.

As before, assume that there are K generations in the family and there are $l_{k}$ different restaurants in generation k. The restaurants correspond to the nodes of DAG D. The customers coming in restaurant j of generation k correspond to parameters $\theta_{ji}^{\left(k\right)}$. Let $\phi_{1},\phi_{2},\ldots ,\phi_{L}$ denote i.i.d. random variables distributed according to the base distribution $G_{0}$, which are dishes from the global menu. To maintain a count of customers and tables, we introduce two notations. We use the notation $n_{jt}^{\left(k\right)}$ to denote the number of customers at table t in the restaurant j of generation k and the notation $m_{jl}^{\left(k\right)}$ to denote the number of tables in the restaurant j of generation k that serve dish l. Marginal counts are represented by dots at the appropriate indices. For example, $m_{j}^{\left(k\right)}$ denotes the count of all the tables (regardless of what dishes being served) in the restaurant j of generation k. We introduce the notation $\psi_{jt}^{\left(k,k\right)}$ to denote the dish served at table t in restaurant j of generation k, chosen from the corresponding layer-1 hyper-restaurant $\left(H_{j}^{\left(k,k\right)}\right)$.

We integrate out random measures $\left\{G_{j}^{\left(k\right)},H_{j}^{\left(k,k\right)},H_{j}^{\left(k,k-1\right)},\ldots ,G_{1}^{\left(0\right)}\right\}$ sequentially. First, we find the conditional distribution of $\theta_{ji}^{\left(k\right)}$ given $\theta_{j1}^{\left(k\right)},\theta_{j2}^{\left(k\right)},\ldots ,\theta_{j,i-1}^{\left(k\right)},\alpha_{j}^{\left(k\right)}$, and $H_{j}^{\left(k,k\right)}$ with $G_{j}^{\left(k\right)}$ integrated out,

(14) $$\theta_{ji}^{\left(k\right)}|\theta_{j1}^{\left(k\right)},\theta_{j2}^{\left(k\right)},\ldots ,\theta_{j,i-1}^{\left(k\right)},\alpha_{j}^{\left(k\right)},H_{j}^{\left(k,k\right)}\sim \sum_{t=1}^{m_{j.}^{\left(k\right)}}\frac{n_{jt}^{\left(k\right)}}{i-1+\alpha_{j}^{\left(k\right)}}\delta_{\psi_{jt}^{\left(k,k\right)}}+\frac{\alpha_{j}^{\left(k\right)}}{i-1+\alpha_{j}^{\left(k\right)}}H_{j}^{\left(k,k\right)},$$

We let $\psi_{jt}^{\left(k,k-1\right)}$ to denote the dish served at table t in the layer-1 hyper-restaurant corresponding to restaurant j of generation k, chosen from the dishes served in the layer-2 hyper-restaurants $\left(H_{j}^{\left(k,k-1\right)}\right)$. Integrating out the hidden measure from the current layer $H_{j}^{\left(k,k\right)}$, the conditional distribution of $\psi_{jt}^{\left(k,k\right)}$ given $\psi_{j1}^{\left(k,k-1\right)},\psi_{j2}^{\left(k,k-1\right)},\ldots ,\psi_{j1}^{\left(k,k\right)},\ldots ,\psi_{j,t-1}^{\left(k,k\right)},\left\{\alpha_{l}^{\left(k-1\right)}:l\in an^{\left(k,1\right)}(j)\right\}$, and the hidden measure from the previous layer, $H_{j}^{\left(k,k-1\right)}$ is given by,

(15) $$\psi_{jt}^{\left(k,k\right)}|\psi_{j1}^{\left(k,k-1\right)},\psi_{j2}^{\left(k,k-1\right)},\ldots ,\psi_{j1}^{\left(k,k\right)},\ldots ,\psi_{j,t-1}^{\left(k,k\right)},\left\{\alpha_{l}^{\left(k-1\right)}:l\in an^{\left(k,1\right)}(j)\right\},H_{j}^{\left(k,k-1\right)}\sim \sum_{l=1}^{M_{j}^{\left(k,k-1\right)}}\frac{m_{jl}^{\left(k,k-1\right)}}{m_{j.}^{\left(k,k-1\right)}+\sum_{l\in an^{\left(k,1\right)}(j)}\alpha_{l}^{\left(k-1\right)}}\delta_{\psi_{jl}^{\left(k,k-1\right)}}+\frac{\sum_{l\in an^{\left(k,1\right)}(j)}\alpha_{l}^{\left(k-1\right)}}{m_{j.}^{\left(k,k-1\right)}+\sum_{l\in an^{\left(k,1\right)}(j)}\alpha_{l}^{\left(k-1\right)}}H_{j}^{\left(k,k-1\right)},$$

where the notation $m_{jl}^{\left(k,k-1\right)}$ denotes the number of tables in layer-1 hyper-restaurant, corresponding to restaurant j of generation k serving the dish l. We denote by $M_{j}^{\left(k,k-1\right)}$ the number of dishes served in the layer-1 hyper-restaurants and by $m_{j}^{\left(k,k-1\right)}$ the total number of tables in the layer-1 hyper-restaurant, corresponding to the restaurant j of generation k. Similarly, integrating out the measure $H_{j}^{\left(k,k-1\right)}$ and introducing the next layer of variables $\psi_{jt}^{\left(k,k-2\right)}$, the conditional distribution of $\psi_{jt}^{\left(k,k-1\right)}$ given $\psi_{j1}^{\left(k,k-2\right)},\psi_{j2}^{\left(k,k-2\right)},\ldots ,\psi_{j1}^{\left(k,k-1\right)},\ldots ,\psi_{j,t-1}^{\left(k,k-1\right)},\left\{\alpha_{l}^{\left(k-2\right)}:l\in an^{\left(k,2\right)}(j)\right\}$, and the hidden measure from the previous layer $H_{j}^{\left(k,k-2\right)}$ is given by,

(16) $$\psi_{jt}^{\left(k,k-1\right)}|\psi_{j1}^{\left(k,k-2\right)},\psi_{j2}^{\left(k,k-2\right)},\ldots ,\psi_{j1}^{\left(k,k-1\right)},\ldots ,\psi_{j,t-1}^{\left(k,k-1\right)},\left\{\alpha_{l}^{\left(k-2\right)}:l\in an^{\left(k,2\right)}\left(j\right)\right\},H_{j}^{\left(k,k-2\right)}\sim \sum_{l=1}^{M_{j}^{\left(k,k-2\right)}}\frac{m_{jl}^{\left(k,k-2\right)}}{m_{j.}^{\left(k,k-2\right)}+\sum_{l\in an^{\left(k,2\right)}(j)}\alpha_{l}^{\left(k-2\right)}}\delta_{\psi_{jl}^{\left(k,k-2\right)}}+\frac{\sum_{l\in an^{\left(k,2\right)}(j)}\alpha_{l}^{\left(k-2\right)}}{m_{j.}^{\left(k,k-2\right)}+\sum_{l\in an^{\left(k,2\right)}(j)}\alpha_{l}^{\left(k-2\right)}}H_{j}^{\left(k,k-2\right)}.$$

As in the stick-breaking representation, we can recursively integrate out hidden measures and eventually arrive at the conditional distribution of $\psi_{jt}^{\left(k,2\right)}$ given $\psi_{j1}^{\left(0\right)},\psi_{j2}^{\left(0\right)},\ldots ,\psi_{j1}^{\left(k,2\right)},\ldots ,\psi_{j,t-1}^{\left(k,2\right)},\left\{\alpha_{l}^{\left(1\right)}:l\in an^{\left(k,k-1\right)}(j)\right\}$, and the probability measure of the root node $G_{1}^{\left(0\right)}$,

(17) $$\psi_{jt}^{\left(k,2\right)}|\psi_{j1}^{\left(0\right)},\psi_{j2}^{\left(0\right)},\ldots ,\psi_{j1}^{\left(k,2\right)},\ldots ,\psi_{j,t-1}^{\left(k,2\right)},\left\{\alpha_{l}^{\left(1\right)}:l\in an^{\left(k,k-1\right)}\left(j\right)\right\},G_{1}^{\left(0\right)}\sim \overset{M_{j}^{\left(k,1\right)}}{\underset{l=1}{\sum }}\frac{m_{jl}^{\left(k,1\right)}}{m_{j.}^{\left(k,1\right)}+\underset{l\in an^{\left(k,k-1\right)}\left(j\right)}{\sum }\alpha_{l}^{\left(1\right)}}\delta_{\psi_{jl}^{\left(0\right)}}+\frac{\sum_{l\in an^{\left(k,k-1\right)}\left(j\right)}\alpha_{l}^{\left(1\right)}}{m_{j.}^{\left(k,1\right)}+\sum_{l\in an^{\left(k,k-1\right)}\left(j\right)}\alpha_{l}^{\left(1\right)}}G_{1}^{\left(0\right)},$$

and the conditional distribution of $\psi_{jt}^{\left(0\right)}$ given $\psi_{j1}^{\left(0\right)},\ldots ,\psi_{j,t-1}^{\left(0\right)},\alpha_{1}^{\left(0\right)}$, and the base measure $G_{0}$,

(18) $$\psi_{jt}^{\left(0\right)}|\psi_{j1}^{\left(0\right)},\ldots ,\psi_{j,t-1}^{\left(0\right)},\alpha_{1}^{\left(0\right)},G_{0}\sim \sum_{l=1}^{L}\frac{m_{l}^{\left(0\right)}}{m_{.}^{\left(0\right)}+\alpha_{1}^{\left(0\right)}}\delta_{\phi_{l}}+\frac{\alpha_{1}^{\left(0\right)}}{m_{.}^{\left(0\right)}+\alpha_{1}^{\left(0\right)}}G_{0},$$

where $m_{l}^{\left(0\right)}$ denotes the number of tables in the original restaurant serving dish l and $m_{.}^{\left(0\right)}$ denotes the total number of tables in the original restaurant. Note that (18) corresponds to the case where the root node is hidden (the same as in HDP). When the root node is not hidden, a similar formula can be derived, which is omitted for simplicity.

### 4.4 The Infinite Limit of Finite Mixture Model

The GDP mixture model can be derived as the infinite limit of a finite mixture model. Let us denote the observations and the mixture component indicator from node j in layer k of DAG D by $x_{ji}^{\left(k\right)}$ and $z_{ji}^{\left(k\right)}$, respectively. Suppose $\beta_{1}^{\left(0\right)}$ is the vector of mixing weights for the root node. Denoting by $\beta_{j}^{\left(k\right)}$ the mixing weights of node j in layer k and by $\nu_{j}^{\left(k,m\right)}$ the corresponding mixing weights for the hidden layer m, with m=2,…,k, we consider a finite mixture version of the proposed GDP,

(19) $$\beta_{1}^{\left(0\right)}|\alpha_{1}^{\left(0\right)}\sim Dir\left(\alpha_{1}^{\left(0\right)}/L,\ldots ,\alpha_{1}^{\left(0\right)}/L\right),\;\nu_{j}^{\left(k,2\right)}|\left\{\alpha_{l}^{\left(1\right)}:l\in an^{\left(k,k-1\right)}\left(j\right)\right\},\beta_{1}^{\left(0\right)}\sim Dir\left(\underset{l\in an^{\left(k,k-1\right)}\left(j\right)}{\sum }\alpha_{l}^{\left(1\right)}\left(\beta_{11}^{\left(0\right)},\ldots ,\beta_{1L}^{\left(0\right)}\right)\right),\;\vdots \;\nu_{j}^{\left(k,k\right)}|\left\{\alpha_{l}^{\left(k,k-1\right)}:l\in an^{\left(k,1\right)}\left(j\right)\right\},\nu_{j}^{\left(k,k-1\right)}\sim Dir\left(\underset{l\in an^{\left(k,1\right)}\left(j\right)}{\sum }\alpha_{l}^{\left(k-1\right)}\left(\nu_{j1}^{\left(k,k-1\right)},\ldots ,\nu_{jL}^{\left(k,k-1\right)}\right)\right),\;\beta_{j}^{\left(k\right)}|\alpha_{j}^{\left(k\right)},\nu_{j}^{\left(k,k\right)}\sim Dir\left(\alpha_{j}^{\left(k\right)}\left(\nu_{j1}^{\left(k,k\right)},\ldots ,\nu_{jL}^{\left(k,k\right)}\right)\right),\;\phi_{l}|G_{0}\sim G_{0},\;z_{ji}^{\left(k\right)}|\beta_{j}^{\left(k\right)}\sim \beta_{j}^{\left(k\right)},\;x_{ji}^{\left(k\right)}|z_{ji}^{\left(k\right)},{\left(\phi_{l}\right)}_{l=1}^{L}\sim F\left(\phi_{z_{ji}^{\left(k\right)}}\right).$$

The distribution of this finite mixture model approaches the GDP mixture model as L→∞. Refer to Section C of the Appendix for the proof. Based on this finite mixture model approximation with a large enough truncation level L, we develop an efficient posterior inference procedure of our model using a Metropolis-within-blocked-Gibbs sampler with a specialized proposal (Director et al., 2017); see Section D of the Appendix for details.

## 5 Simulations

**Figure 7:**
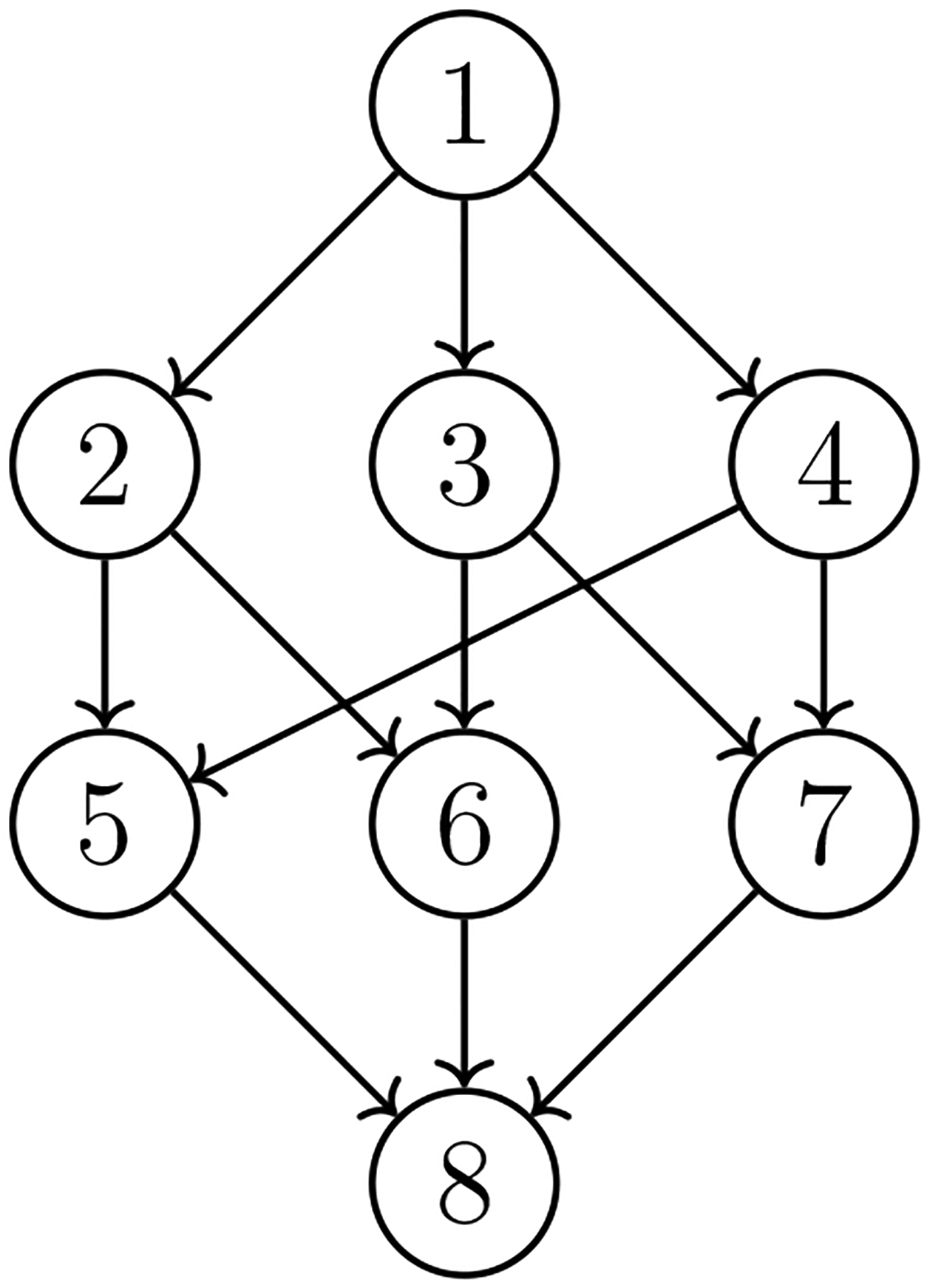
The DAG of experimental groups.

Our simulations are designed to mimic the motivating application where we have 8 experimental groups, whose relationships are represented by the DAG in Figure 7

We generated data within each of the 8 groups from a four-component mixture of bivariate Gaussian distributions with different covariance matrices for each group. We drew the DP concentration parameters $\alpha_{j}$’s for the different groups from their prior distribution (10) respecting the DAG in Figure 7 with $\alpha_{0}=5$. The weights of the finite mixture model corresponding to the different groups were drawn using (19) and the same DAG. The true cluster indicators of each group were sampled from a multinomial distribution with probabilities equal to the mixture weights. Using these true cluster indices for each group, samples were drawn from the Gaussian distribution with the cluster-specific mean and group-specific covariance matrix, given in Tables 4 and 5, respectively, in Section E of the Appendix. Refer to the same section in the Appendix for more details on our simulation strategy. In our Gibbs sampler, the truncation level of the finite mixture model was set to L=10, and the base measure for GDP, $G_{0}$, was specified as the normal-inverse-Wishart distribution, $𝒩ℐ𝒲\left(0,0.01,I_{2},2\right)$. Upon the completion of the Gibbs sampler, the clusters were estimated by using the least squares criterion (Dahl, 2006), and they were compared with the true cluster labels for evaluation. We considered various sample sizes in each group, which are summarized in Table 1. In all cases, we ran 15,000 iterations of our Gibbs sampler and after discarding the first 5,000 samples as burn-in, we retained every 10th iteration of posterior samples.

**Figure 8:**
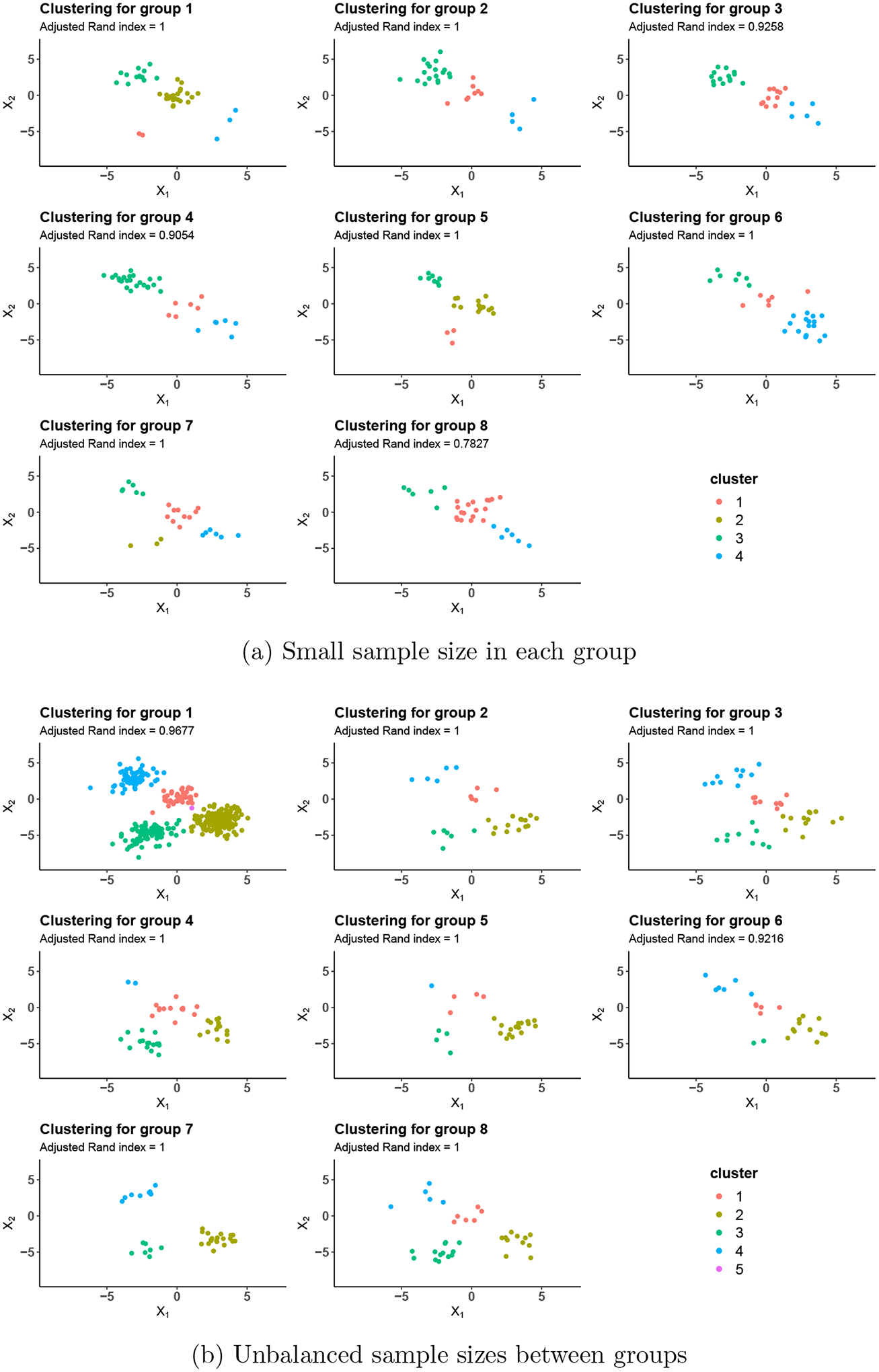
Clustering performance of GDP for different sample sizes. The colors indicate the estimated clusters by GDP. Adjusted Rand index is reported at the top of each panel.

The clustering results of GDP for small and unbalanced sample sizes are visualized in Figure 8. The remaining clustering plots are shown in Appendix E. Across different sample sizes, the proposed GDP was able to identify the clusters within each group with very good accuracy and was able to link clusters across non-exchangeable groups.

We also looked at the clustering performance of GDP under a more difficult scenario. The simulation details and clustering results are shown in Appendix E. Since HDP is a special case of the proposed GDP, we compared the two methods for this difficult scenario. We also compared the clustering performance of GDP with k-means, a widely used non-Bayesian clustering technique. The number of clusters in k-means was taken to be the truncation level of our GDP. All simulations were replicated 50 times.

**Figure 9:**
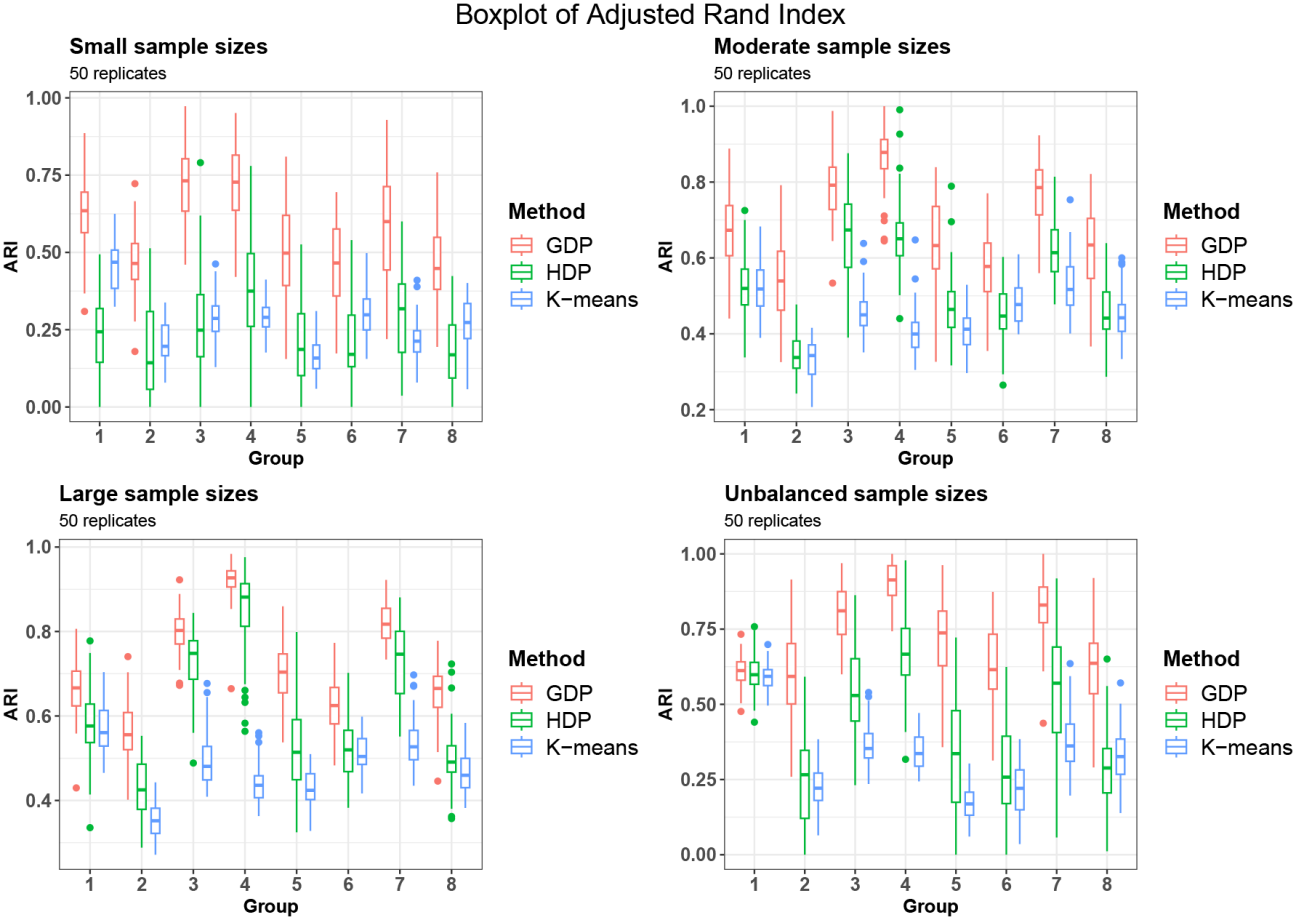
The boxplots of the adjusted Rand indices for GDP, HDP, and k-means for all sample sizes.

GDP significantly outperformed both HDP and k-means. For example, the boxplots of adjusted Rand indices (Hubert and Arabie, 1985; higher is better) for the different methods are shown in Figure 9. It is evident that the adjusted Rand indices of GDP were almost uniformly higher than those of HDP because HDP was not able to handle non-exchangeable groups. Similarly, the higher adjusted Rand indices of GDP indicated its superior clustering performance over the k-means algorithm. Moreover, k-means algorithm does not allow sharing of relevant clusters across the groups.

We further explored additional simulations for grouped data characterized by dependencies that can be represented by a known DAG, beyond those that mimick our motivational application. Specifically, we investigated time-dependent grouped data, which can be represented using an autoregressive (AR) model. Such an AR model may be analyzed using the GDP. The proposed model, simulation details, and clustering results for various number of time points (groups) are shown in Appendix Section F. In summary, our model was able to identify the clusters within each group and link them across groups with good accuracy.

## 6 Real Data Analysis

With the advancement of next-generation sequencing techniques in recent years, it is now possible to molecularly characterize individual cells, which may provide valuable insights into complex biological systems, ranging from cancer genomics to diverse microbial communities (Hwang et al., 2018). Colorectal cancer is the third most common type of cancer after breast and lung cancers. It is known that the mutation of tumor-suppressor gene Apc is an initial step in most colorectal tumors (Morin et al., 1997). In addition, numerous studies have been conducted to understand the effect of high-fat vs low-fat diet on gene expressions (Jump and Clarke, 1999; Bouchard-Mercier et al., 2013; Fan et al., 2020). We are motivated by a study that aimed to investigate how diet, genotype, and treatment with a new cancer prevention drug (AdipoRon) against placebo interacted to influence the expression of genes in intestinal crypt and tumor cells. The experiments started from a baseline group where the mice were genetically wild-type, fed with a normal diet, and treated with placebo. Then to understand the main effects of genotype, diet, and cancer treatment on stem cell gene expression, the experimenters introduced three new groups of mice, each differing from the baseline group by exactly one factor (Apc knock-out, high-fat diet, or new cancer treatment AdipoRon). To determine the two-way interaction effects, three additional groups of mice were studied, each of which differed from the baseline group by two factors (e.g., mice with Apc knock-out, high-fat diet, and placebo). Lastly, for a three-way interaction, the experimenters introduced the eighth group of mice with Apc knock-out, a high-fat diet, and the new treatment AdipoRon. By design, these 8 experimental groups are non-exchangeable and their relationships can be delineated by the DAG in Figure 7. The goal of this analysis is to identify potential intestinal molecular subtypes within each experimental group while allowing information to be shared across these non-exchangeable groups with the proposed GDP model. For illustration, we randomly sampled 100 cells from each of the eight groups. The scRNA-seq data were pre-processed following standard procedure as outlined by Hao et al. (2021) using the R package Seurat. The data was log-normalized and scaled such that the mean expression across cells was 0 and the variance across cells was 1. As a common practice in single-cell data analyses, the uniform manifold approximation and projection (UMAP) (McInnes et al. 2018) was used to reduce the data to two dimensions. We considered the truncation level, L=30, and the same base probability measure, $G_{0}$, as in the simulations. Furthermore, we have considered several choices of the truncation level of the GDP, which shows our method is relatively robust for L≥30; see Appendix G for details. We ran four parallel chains of the Gibbs sampler for 50,000 iterations. To monitor the convergence of the sampler, we drew the traceplots of the log-likelihood for each of the four chains, after discarding the initial 35, 000 samples and thinning the samples by a factor of 15, which indicated no lack of convergence of our sampler. We pooled the Monte Carlo samples across different chains for posterior inference. We compared the clustering performance with that obtained from HDP on the same data.

**Figure 10:**
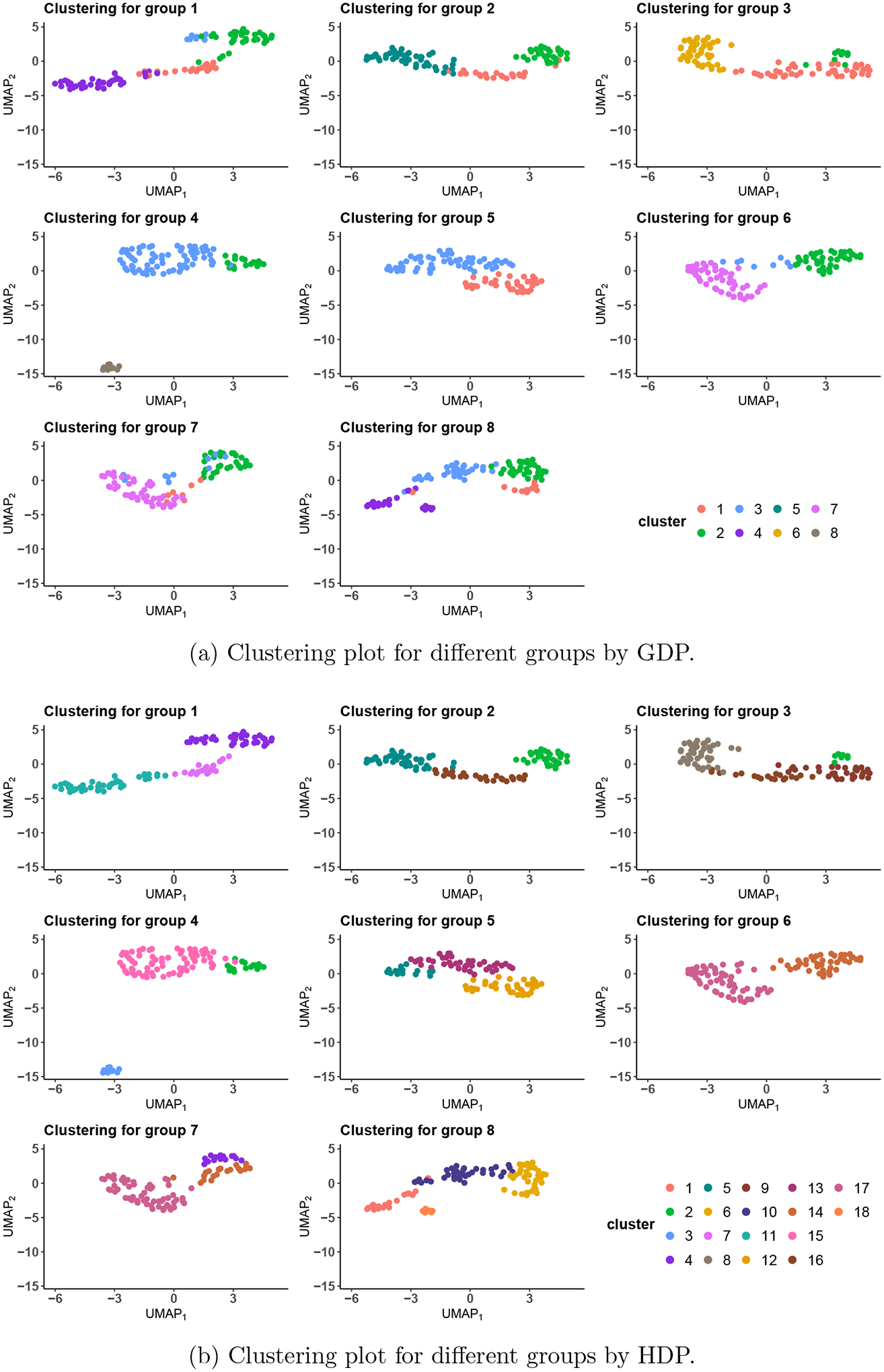
Clustering of the group-specific single-cell data whose dimensions are reduced to 2 by UMAP by (a) GDP and (b) HDP.

The estimated clusters from GDP and HDP are shown in Figures 10a and 10b, respectively. As shown in Table 3 in Section D of the Appendix, group 1 is the wild-type group receiving the placebo and a normal diet. Each of group 2, 3, and 4 are obtained from group 1 by changing the three factors one at a time, and hence shares some similar clusters with group 1. Group 4 is similar to the baseline group 1 but with the Apc gene knocked out. The corresponding clustering plot (Figure 10a) of GDP indicates that the Apc knock-out group seems to exhibit more heterogeneity of cells (suggesting possibly new cellular subtypes) as compared to the wild-type group. Group 5 is the Apc knock-out group receiving a high-fat diet and the placebo. The clustering plot shows some resemblance with its parent groups (groups 2 and 4) but with the absence of some parental clusters. Groups 6 and 7 show similar clustering patterns, indicating possibly similar impact of changing the corresponding factors from their parent groups. Groups 7 and 8 correspond to the Apc knock-out group receiving the new treatment and fed with a normal and high-fat diet, respectively. It can be seen that the high-fat diet group appears to have greater molecular heterogeneity than the normal diet group. The Figure 10b, on the other hand, clearly shows that HDP fails to capture meaningful clusters across the non-exchangeable groups, i.e., some points that seemingly belong to the same cluster are assigned different labels across groups. To quantify the difference between GDP and HDP, we computed several internal clustering validation measures; see Liu et al., 2010 for a review of several such measures. Table 2 compares the Calinski-Harabasz, Davies–Bouldin, and Silhouette Index between GDP and HDP. Clearly, all of them indicate the superior clustering performance of GDP over HDP.

## 7 Discussion

We have introduced the GDP as a graph-based stochastic process for modeling dependent random measures that are linked by a DAG. We have also introduced the corresponding infinite mixture model and presented how the GDP mixture model can be used for clustering grouped data with non-exchangeable groups. We provided different representations of the GDP including a novel hypergraph representation of the original process. The posterior inference was relatively straightforward. We illustrated our method using both simulations and an application to a real grouped scRNA-seq data set.

There are a few possible future directions for this work. First, it may be possible to replace the DAG in our GDP with an undirected or chain graph. The challenge is to define the joint distribution over a set of random measures given the graph where the convenient DAG factorization no longer applies. Second, it may also be possible to learn the DAG structure instead of assuming it is known, which may require independent realizations of the GDP. In theory, if there are replicates from the underlying joint distribution of random probability measures, it is possible to identify the underlying DAG up to its Markov equivalent class. In that case, we can either consider a uniform prior for DAG D,p(D)∝1 or a prior that penalizes the graph complexity, $p(D)\propto \theta^{\left|D\right|}$ where θ∈(0,1) and |D| is the number of edges in D. Then the posterior inference can be carried out by searching the DAG space via MCMC with edge addition, deletion, and reversal moves; see e.g., Section 2.4 of Choi et al. (2020). For multivariate data, a DAG may be uniquely identifiable under certain conditions such as non-Gaussianity (Shimizu et al., 2006). In the proposed construction of the GDP, we have assumed that the random probability measures corresponding to each node is non-Gaussian, i.e., a Dirichlet process. Therefore, it might be possible to extend the ideas of Shimizu et al., 2006 in our setup, replacing random variables corresponding to each node with random probability measures for the unique identifiability of the underlying DAG. Third, the proposed construction of the GDP can be extended for other processes including the Pitman-Yor process and more generally the completely random measures and normalized random measures. For the proposed *Graphical Dirichlet Process*, we have assumed that $G_{j}$ is a Dirichlet Process with the base measure being a weighted mixture of the measures of the corresponding parent nodes $\left(\left\{G_{l}:l\in pa(j)\right\}\right)$, i.e., $G_{j}|\alpha_{j},\left\{G_{l}:l\in pa(j)\right\}\sim DP\left(\alpha_{j},\sum_{l\in pa(j)}\pi_{jl}G_{l}\right)$. This can, by construction, be replaced by the Pitman-Yor process i.e., $G_{j}|\alpha_{j},\sigma_{j},\left\{G_{l}:l\in pa(j)\right\}\sim 𝒫𝒴\left(\alpha_{j},\sigma_{j},\sum_{l\in pa(j)}\pi_{jl}G_{l}\right)$, where 𝒫𝒴(a,b,π) denotes a Pitman-Yor process with concentration parameter a, discount parameter b>-a, and base measure π, which leads to a *Graphical Pitman-Yor Process*. Alternatively, one may consider a *Graphical Gamma Process* by assuming $G_{j}|\alpha_{j},\left\{G_{l}:l\in pa(j)\right\}\sim \Gamma P\left(\alpha_{j},\sum_{l\in pa(j)}\pi_{jl}G_{l}\right)$, where ΓP(a,π) is a Gamma process with concentration parameter a, and base measure π. One caveat though is that the proposed hypergraph representation of the proposed GDP (Theorem 3) relies on the fact that a Dirichlet mixture of DPs is a DP. Therefore, for other processes, to get an equivalent hypergraph representation the choice of the hyperpriors must be redesigned, which could be an interesting future direction..

## Supplementary Material

1

## Figures and Tables

**Table 1:** The sample sizes for the different groups that were used to simulate the data.

Sample sizes	Groups							
	1	2	3	4	5	6	7	8
small	40	30	30	35	25	30	25	30
moderate	80	70	70	75	83	88	92	88
large	150	160	180	170	155	175	185	145
unbalanced	350	30	40	45	25	25	35	35

**Table 2:** Different measures of internal clustering for GDP and HDP. Higher values of Calinski-Harabasz Index and Silhouette Index indicates better clustering. Lower values of Davies–Bouldin Index indicate well separated clusters.

	Calinski-Harabasz Index	Davies–Bouldin Index	Silhouette Index
GDP	418.518	1.559	0.198
HDP	225.327	4.868	−0.044
