# Supplementary material for: Graphical Dirichlet Process for Clustering Non-Exchangeable Grouped Data
Source: J Mach Learn Res. Author manuscript; Available in PMC 2024 Dec 17. (PMC11650374)
Supplement: 1 [file NIHMS2041132-supplement-1.pdf]

Appendix A. Proof of the Hypergraph Representation

We prove Theorem 3 (the hypergraph representation of the proposed GDP) of the main manuscript in the case of our motivational problem where we have 8 groups. Note that the proof for any general DAG follows in a similar fashion by repeated application of the two lemmas in Section 3.3 of the main manuscript and properties of gamma and Dirichlet distributions, which, however, requires more involved bookkeeping of the corresponding random distributions and hence is omitted. Our proof also illustrates how the random distribution of any particular node of the DAG is related to the root node through a number of hidden random measures, which shows the clustering property of our model. In our motivating example, each group corresponds to a combination of treatment, diet, and genotype, as summarized in Table 3 in Section D of the Appendix. The underlying DAG for the problem is given in Figure 7 of the main manuscript where group 1 is the root node, groups 2-4 are the layer-1 nodes, groups 5-7 are the layer-2 nodes, and group 8 is the layer-3 node. For ease of notation, instead of using $G_1^{(0)}$ and $\alpha_1^{(0)}$ to denote the random measure and the concentration parameter of the root node, we use simply G_1 and α_1 instead; similarly for all the other nodes. Using these simplified notations, Figures 11a and 11b show the relationships among the group-specific random measures and concentration parameters according to Figure 7 of the main manuscript.

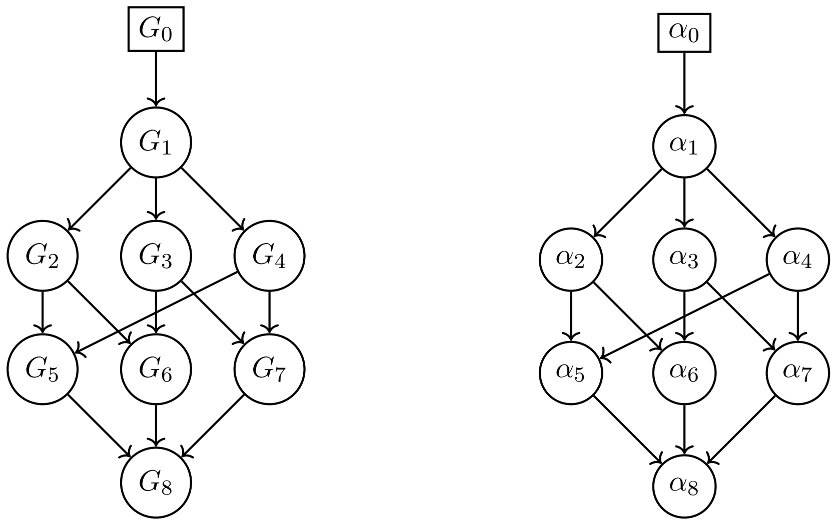

(a) (b)

Figure 11: The DAG of the (a) random measures G_j's and (b) concentration parameters α_j's.

The proposed GDP mixture model for this problem is given hierarchically as,

$$\alpha_1 \sim Gamma(\alpha_0, 1), \qquad G_1 \sim DP(\alpha_1, G_0),$$

$$
\begin{aligned}
\alpha_j &\sim Gamma(\alpha_1, 1), \;\; j = 2,3,4, & G_j &\sim DP(\alpha_j, G_1), \;\; j = 2,3,4,\\
\alpha_5 &\sim Gamma(\alpha_2 + \alpha_4, 1), & G_5 &\sim DP(\alpha_5, \pi_1 G_2 + (1-\pi_1) G_4),\\
& & \pi_1 &\sim Beta(\alpha_2, \alpha_4),\\
\alpha_6 &\sim Gamma(\alpha_2 + \alpha_3, 1), & G_6 &\sim DP(\alpha_6, \pi_2 G_2 + (1-\pi_2) G_3),\\
& & \pi_2 &\sim Beta(\alpha_2, \alpha_3),\\
\alpha_7 &\sim Gamma(\alpha_3 + \alpha_4, 1), & G_7 &\sim DP(\alpha_7, \pi_3 G_3 + (1-\pi_3) G_4),\\
& & \pi_3 &\sim Beta(\alpha_3, \alpha_4),\\
\alpha_8 &\sim Gamma(\alpha_5 + \alpha_6 + \alpha_7, 1), & G_8 &\sim DP(\alpha_8, \gamma_1 G_5 + \gamma_2 G_6 + \gamma_3 G_7),\\
& & \boldsymbol{\gamma} = (\gamma_1,\gamma_2,\gamma_3) &\sim Dir(\alpha_5, \alpha_6, \alpha_7),\\
\theta_{ji} \mid G_j &\overset{ind}{\sim} G_j,\\
x_{ji} \mid \theta_{ji} &\overset{ind}{\sim} F(\theta_{ji}), & & i = 1,\ldots,n_j, \;\; j = 1,\ldots,8. \qquad (20)
\end{aligned}
$$

Now, from Theorem 3, we have the following hypergraph representation, which we are going to prove,

$$
\begin{aligned}
\alpha_1 &\sim Gamma(\alpha_0, 1), & G_1 &\sim DP(\alpha_1, G_0),\\
\alpha_j &\sim Gamma(\alpha_1, 1), \;\; j = 2,3,4, & G_j &\sim DP(\alpha_j, G_1), \;\; j = 2,3,4,\\
\alpha_5 &\sim Gamma(\alpha_2 + \alpha_4, 1), & G_5 &\sim DP(\alpha_5, H_1)\\
& & H_1 &\sim DP(\alpha_2 + \alpha_4, G_1),\\
\alpha_6 &\sim Gamma(\alpha_2 + \alpha_3, 1), & G_6 &\sim DP(\alpha_6, H_2),\\
& & H_2 &\sim DP(\alpha_2 + \alpha_3, G_1),\\
\alpha_7 &\sim Gamma(\alpha_3 + \alpha_4, 1), & G_7 &\sim DP(\alpha_7, H_3),\\
& & H_3 &\sim DP(\alpha_3 + \alpha_4, G_1),\\
\alpha_8 &\sim Gamma(\alpha_5 + \alpha_6 + \alpha_7, 1), & G_8 &\sim DP(\alpha_8, H_4),\\
& & H_4 &\sim DP(\alpha_5 + \alpha_6 + \alpha_7, H^*),\\
& & H^* &\sim DP(2(\alpha_2 + \alpha_3 + \alpha_4), G_1),\\
\theta_{ji} \mid G_j &\overset{ind}{\sim} G_j,\\
x_{ji} \mid \theta_{ji} &\overset{ind}{\sim} F(\theta_{ji}), & & i = 1,\ldots,n_j, \;\; j = 1,\ldots,8.
\end{aligned} \qquad (21)
$$

Proof

Note that the random measures G_2, G_3, and G_4 are the layer-1 nodes. Their relationships to the root node G_1 are the same as those in an HDP. We shall consider the relationships of the random measures of the layer-2 and layer-3 nodes (i.e., G_5, G_6, G_7, and G_8) to the root node. Let $H_1 = \pi_1 G_2 + (1 - \pi_1) G_4$ where $G_2 \sim DP(\alpha_2, G_1)$ and $G_4 \sim DP(\alpha_4, G_1)$ independently. Let $A_1, A_2, \ldots, A_r$ be a finite measurable partition of the sample space Θ. Then by the definition of DP, we have

$$
\begin{aligned}
(G_2(A_1), G_2(A_2), \ldots, G_2(A_r)) &\sim Dir\left(\alpha_2 G_1(A_1), \alpha_2 G_1(A_2), \ldots, \alpha_2 G_1(A_r)\right),\\
(G_4(A_1), G_4(A_2), \ldots, G_4(A_r)) &\sim Dir\left(\alpha_4 G_1(A_1), \alpha_4 G_1(A_2), \ldots, \alpha_4 G_1(A_r)\right),
\end{aligned}
$$

which are conditionally independent given α_2, α_4 and G_1. As $\pi_1 \sim Beta(\alpha_2, \alpha_4)$ independently of G_2 and G_4, using Theorem 1, we have that, given α_2, α_4 and G_1,

$$
\pi_1 \left(G_2(A_1), \ldots, G_2(A_r)\right) + (1 - \pi) \left(G_4(A_1), \ldots, G_4(A_r)\right)
$$

$$
\begin{aligned}
& && \sim Dir((\alpha_2+\alpha_4)(G_1(A_1),\ldots,G_1(A_r))) \\
& \Rightarrow (H_1(A_1),\ldots,H_1(A_r)) \mid \alpha_2,\alpha_4,G_1 && \sim Dir((\alpha_2+\alpha_4)(G_1(A_1),\ldots,G_1(A_r))) \\
& \Rightarrow H_1 \mid \alpha_2,\alpha_4,G_1 && \sim DP(\alpha_2+\alpha_4,G_1)
\end{aligned}
$$

Thus, we have

$$
\begin{aligned}
G_5 \mid H_1,\alpha_5 &\sim DP(\alpha_5,H_1), \\
H_1 \mid \alpha_2,\alpha_4,G_1 &\sim DP(\alpha_2+\alpha_4,G_1).
\end{aligned} \tag{22}
$$

Similarly, the other layer-2 measures G_6 and G_7 have the following representations:

$$
\begin{aligned}
G_6 \mid H_2,\alpha_6 &\sim DP(\alpha_6,H_2), \\
H_2 \mid \alpha_2,\alpha_3,G_1 &\sim DP(\alpha_2+\alpha_3,G_1),
\end{aligned} \tag{23}
$$

and,

$$
\begin{aligned}
G_7 \mid H_3,\alpha_7 &\sim DP(\alpha_7,H_3), \\
H_3 \mid \alpha_3,\alpha_4,G_1 &\sim DP(\alpha_3+\alpha_4,G_1),
\end{aligned} \tag{24}
$$

where $H_2 = \pi_2 G_2 + (1-\pi_2)G_3$ and $H_3 = \pi_3 G_3 + (1-\pi_3)G_4$.

Let $H_4 = \gamma_1 G_5 + \gamma_2 G_6 + \gamma_3 G_7$ and $\boldsymbol{\gamma} = (\gamma_1,\gamma_2,\gamma_3) \sim Dir(\alpha_5,\alpha_6,\alpha_7)$. Since G_5, G_6, and G_7 are conditionally independent given G_2, G_3, and G_4, they are also independent given H_1, H_2, and H_3. Therefore, we have,

$$
\begin{aligned}
G_5 \mid \alpha_5,H_1 &\sim DP(\alpha_5,H_1), \\
G_6 \mid \alpha_6,H_2, &\sim DP(\alpha_6,H_2), \\
G_7 \mid \alpha_7,H_3 &\sim DP(\alpha_7,H_3).
\end{aligned}
$$

For any finite measurable partition $A_1, A_2, \ldots, A_r$ of Θ, from Theorem 2, we have

$$
\begin{aligned}
& (H_4(A_1),\ldots,H_4(A_r)) \mid \alpha_5,\alpha_6,\alpha_7,H_1,H_2,H_3 \\
= \;& \gamma_1\left(G_5(A_1),\ldots,G_5(A_r)\right) + \gamma_2\left(G_6(A_1),\ldots,G_6(A_r)\right) + \gamma_3\left(G_7(A_1),\ldots,G_7(A_r)\right) \\
& \sim Dir\left((\alpha_5 H_1 + \alpha_6 H_2 + \alpha_7 H_3)(A_1),\ldots,(\alpha_5 H_1 + \alpha_6 H_2 + \alpha_7 H_3)(A_r)\right) \\
& \equiv Dir\left(\alpha^*\left(\left(\frac{\alpha_5}{\alpha^*}H_1 + \frac{\alpha_6}{\alpha^*}H_2 + \frac{\alpha_7}{\alpha^*}H_3\right)(A_1),\ldots,\left(\frac{\alpha_5}{\alpha^*}H_1 + \frac{\alpha_6}{\alpha^*}H_2 + \frac{\alpha_7}{\alpha^*}H_3\right)(A_r)\right)\right) \\
& \equiv Dir\left(\alpha^*\left(H^*(A_1),\ldots,H^*(A_r)\right)\right) \\
& \Rightarrow H_4 \mid \alpha^*, H^* \sim DP(\alpha^*,H^*),
\end{aligned} \tag{25}
$$

where $\alpha^* = \alpha_5+\alpha_6+\alpha_7$ and $H^* = \frac{\alpha_5}{\alpha^*}H_1 + \frac{\alpha_6}{\alpha^*}H_2 + \frac{\alpha_7}{\alpha^*}H_3$. Note that α_5, α_6, and α_7 are independent gamma random variables conditionally on $\alpha_2,\alpha_3,\alpha_4$ with shape parameters $\alpha_2+\alpha_4, \alpha_2+\alpha_3$, and $\alpha_3+\alpha_4$, respectively. Thus,

$$
\left(\frac{\alpha_5}{\alpha^*},\frac{\alpha_6}{\alpha^*},\frac{\alpha_7}{\alpha^*}\right) \mid \alpha_2,\alpha_3,\alpha_4 \sim Dir(\alpha_2+\alpha_4,\alpha_2+\alpha_3,\alpha_3+\alpha_4) \tag{26}
$$

Thus, given $G_1, G_2, G_3, G_4, \alpha_2, \alpha_3, \alpha_4$, and from Eqs. (22–24), and Eq. (26), using Theorem 2 and using a similar measurable finite partition of Θ argument, we have,

$$
H^* \mid \alpha_2,\alpha_3,\alpha_4,G_1 \sim DP(2(\alpha_2+\alpha_3+\alpha_4),G_1), \tag{27}
$$

which completes the proof. ■

Appendix B. Proof of Lemma 1 and Lemma 2

B.1 Proof of Lemma 1

Lemma 1 (**Sethuraman, 1994**) *Let $\boldsymbol{\alpha}_1 = (\alpha_{11}, \alpha_{12}, \ldots, \alpha_{1k})$ and $\boldsymbol{\alpha}_2 = (\alpha_{21}, \alpha_{22}, \ldots, \alpha_{2k})$ be k-dimensional vectors with $\alpha_{ij} > 0 \ \forall \ j = 1, 2, \ldots, k, \ i = 1, 2$. Let $\boldsymbol{X}_1$ and $\boldsymbol{X}_2$ be independent k-dimensional random vectors distributed as Dirichlet distribution with parameters $\boldsymbol{\alpha}_1$ and $\boldsymbol{\alpha}_2$, respectively. Let $\alpha_{1\cdot} = \sum_{j=1}^{k} \alpha_{1j}$ and $\alpha_{2\cdot} = \sum_{j=1}^{k} \alpha_{2j}$. Let π be independent of $\boldsymbol{X}_1$ and $\boldsymbol{X}_2$ and have a beta distribution $Beta\,(\alpha_{1\cdot}, \alpha_{2\cdot})$. Then the distribution of $\pi \boldsymbol{X}_1 + (1 - \pi)\,\boldsymbol{X}_2$ is the Dirichlet distribution with parameter $\boldsymbol{\alpha}_1 + \boldsymbol{\alpha}_2$.*

Proof Let $T_i \overset{ind}{\sim} Gamma\,(\alpha_{1i}, \lambda)\,, \ i = 1, 2, \ldots, k$ and $S_i \overset{ind}{\sim} Gamma\,(\alpha_{2i}, \lambda)\,, \ i = 1, 2, \ldots, k$ independently of T_i, where $\lambda > 0$. Let $T = \sum_{i=1}^{k} T_i$ and $S = \sum_{i=1}^{k} S_i$. We know from the reproductive property of independent gamma distributions that $T \sim Gamma\left(\sum_{i=1}^{k} \alpha_{1i}, \lambda\right) \equiv Gamma\,(\alpha_{1\cdot}, \lambda)$ and $S \sim Gamma\left(\sum_{i=1}^{k} \alpha_{2i}, \lambda\right) \equiv Gamma\,(\alpha_{2\cdot}, \lambda)$ independently of T. Define

$$\boldsymbol{X}_1 := \left(\frac{T_1}{T}, \frac{T_2}{T}, \ldots, \frac{T_k}{T}\right), \quad \boldsymbol{X}_2 := \left(\frac{S_1}{S}, \frac{S_2}{S}, \ldots, \frac{S_k}{S}\right), \quad \text{and} \quad \pi := \frac{T}{T+S}.$$

It is easy to see that $\boldsymbol{X}_1 \sim Dir(\alpha_{11}, \ldots, \alpha_{1k})$ is independent of $\boldsymbol{X}_2 \sim Dir(\alpha_{21}, \ldots, \alpha_{2k})$, and that $\pi \sim Beta\,(\alpha_{1\cdot}, \alpha_{2\cdot})$. We now need to show that π as defined above is indeed independent of $\boldsymbol{X}_1$ and $\boldsymbol{X}_2$ as required by the lemma. For any fixed $\alpha_{11}, \ldots, \alpha_{1k}$, we have that $\sum_{i=1}^{k} T_i$ is a complete and sufficient statistic for λ. Because $\boldsymbol{X}_1 \sim Dir(\alpha_{11}, \ldots, \alpha_{1k})$ is ancillary for λ, by the Basu's theorem (Basu, 1955), we have that $\boldsymbol{X}_1$ is independent of $\sum_{i=1}^{k} T_i = T$. Furthermore, due to the independence of S_i and T_i, $i = 1, \ldots, k$, $\boldsymbol{X}_1$ is independent of S, and, therefore, $\boldsymbol{X}_1$ is independent of $\pi = \frac{T}{T+S}$. Similarly, $\boldsymbol{X}_2$ is also independent of π. Then,

$$\begin{aligned} \pi \boldsymbol{X}_1 + (1 - \pi)\,\boldsymbol{X}_2 &= \frac{T}{T+S}\left(\frac{T_1}{T}, \frac{T_2}{T}, \ldots, \frac{T_k}{T}\right) + \frac{S}{T+S}\left(\frac{S_1}{S}, \frac{S_2}{S}, \ldots, \frac{S_k}{S}\right) \\ &= \left(\frac{T_1 + S_1}{T+S}, \frac{T_2 + S_2}{T+S}, \ldots, \frac{T_k + S_k}{T+S}\right) \sim Dir\,(\boldsymbol{\alpha}_1 + \boldsymbol{\alpha}_2)\,, \end{aligned}$$

because $T_i + S_i \overset{ind}{\sim} Gamma(\alpha_{1i} + \alpha_{2i}, \lambda) \ \ i = 1, 2, \ldots, k$ and $T + S \sim Gamma(\alpha_{1\cdot} + \alpha_{2\cdot}, \lambda)$. ■

B.2 Proof of Lemma 2

Lemma 2 *Let $\boldsymbol{\alpha}_1, \boldsymbol{\alpha}_2, \ldots, \boldsymbol{\alpha}_L$ be k-dimensional vectors where $\boldsymbol{\alpha}_i = (\alpha_{i1}, \ldots, \alpha_{ik})$ with $\alpha_{ij} > 0 \ \forall \ j = 1, 2, \ldots, k, \ i = 1, 2, \ldots, L$. Let $\boldsymbol{X}_1, \boldsymbol{X}_2, \ldots, \boldsymbol{X}_L$ be independent k-dimensional random vectors distributed as Dirichlet distribution with parameters $\boldsymbol{\alpha}_1, \boldsymbol{\alpha}_2, \ldots, \boldsymbol{\alpha}_L$, respectively. Let $\alpha_{i\cdot} = \sum_{j=1}^{k} \alpha_{ij}, \ \ i = 1, 2, \ldots, L$. Let $\boldsymbol{\pi} = (\pi_1, \pi_2, \ldots, \pi_L)$ be independent of $\boldsymbol{X}_1, \boldsymbol{X}_2, \ldots, \boldsymbol{X}_L$ and have a Dirichlet distribution $Dir\,(\alpha_{1\cdot}, \alpha_{2\cdot}, \ldots, \alpha_{L\cdot})$. Then the distribution of $\sum_{i=1}^{L} \pi_i \boldsymbol{X}_i$ is the Dirichlet distribution with parameter $\sum_{i=1}^{L} \boldsymbol{\alpha}_i$.*

Proof The proof is similar to that of Appendix B.1. By noting that

$$\boldsymbol{\pi} \sim Dir\,(\alpha_{1\cdot}, \alpha_{2\cdot}, \ldots, \alpha_{L\cdot}) \overset{d}{=} \left(\frac{\gamma_1}{\gamma}, \frac{\gamma_2}{\gamma}, \ldots, \frac{\gamma_L}{\gamma}\right),$$

where $\gamma_i \overset{ind}{\sim} Gamma(\alpha_{i\cdot}, \lambda), \ i = 1, 2, \ldots, L$ and $\gamma = \sum_{i=1}^{L} \gamma_i \sim Gamma\left(\sum_{i=1}^{L} \alpha_{i\cdot}, \lambda\right)$. The remaining proof follows from standard properties of Dirichlet distributions and mimics the proof of

Appendix B.1. ■

Appendix C. Proof of the Infinite Limit of Finite Mixture Model

The GDP mixture model can be derived as the infinite limit of a finite mixture model. Let us denote the observations and the mixture component indicator from node j in layer k of DAG D by $x_{ji}^{(k)}$ and $z_{ji}^{(k)}$, respectively. Let $\boldsymbol{\beta}_1^{(0)}$ be the vector of mixing weights for the root node. Denoting by $\boldsymbol{\beta}_j^{(k)}$ the mixing weights of node j in layer k and by $\nu_j^{(k,m)}$ the corresponding mixing weights for the hidden layer m, with $m = 2, \ldots, k$, we have

$$
\begin{aligned}
\boldsymbol{\beta}_1^{(0)} \mid \alpha_1^{(0)} &\sim Dir\left(\alpha_1^{(0)}/L, \ldots, \alpha_1^{(0)}/L\right), \\
\boldsymbol{\nu}_j^{(k,2)} \mid \{\alpha_l^{(1)} : l \in an^{(k,k-1)}(j)\}, \boldsymbol{\beta}_1^{(0)} &\sim Dir\left(\sum_{l \in an^{(k,k-1)}(j)} \alpha_l^{(1)} \left(\beta_{11}^{(0)}, \ldots, \beta_{1L}^{(0)}\right)\right), \\
&\vdots \\
\boldsymbol{\nu}_j^{(k,k)} \mid \{\alpha_l^{(k,k-1)} : l \in an^{(k,1)}(j)\}, \boldsymbol{\nu}_j^{(k,k-1)} &\sim Dir\left(\sum_{l \in an^{(k,1)}(j)} \alpha_l^{(k-1)} \left(\nu_{j1}^{(k,k-1)}, \ldots, \nu_{jL}^{(k,k-1)}\right)\right), \\
\boldsymbol{\beta}_j^{(k)} \mid \alpha_j^{(k)}, \boldsymbol{\nu}_j^{(k,k)} &\sim Dir\left(\alpha_j^{(k)} \left(\nu_{j1}^{(k,k)}, \ldots, \nu_{jL}^{(k,k)}\right)\right), \\
\phi_l \mid G_0 &\sim G_0, \\
z_{ji}^{(k)} \mid \boldsymbol{\beta}_j^{(k)} &\sim \boldsymbol{\beta}_j^{(k)}, \\
x_{ji}^{(k)} \mid z_{ji}^{(k)}, (\phi_l)_{l=1}^L &\sim F\left(\phi_{z_{ji}^{(k)}}\right).
\end{aligned}
\tag{28}
$$

Proof Consider the random probability measure

$$
G_1^{(0)\,L} = \sum_{l=1}^L \beta_{1l}^{(0)} \delta_{\phi_l}.
$$

Ishwaran and Zarepour, 2002 shows that for every measurable function g, integrable with respect to G_0, we have, given $\alpha_1^{(0)}$, as $L \to \infty$

$$
\int g(\theta) dG_1^{(0),L}(\theta) \overset{\mathcal{D}}{\to} \int g(\theta) dG_1^{(0)}(\theta).
$$

Further, consider

$$
\begin{aligned}
G_j^{(k)\,L} &= \sum_{l=1}^L \beta_{jl}^{(k)} \delta_{\phi_l}, \\
H_j^{(k,m)\,L} &= \sum_{l=1}^L \nu_{jl}^{(k,m)} \delta_{\phi_l}, \quad m = 2, \ldots, k.
\end{aligned}
$$

Let $(A_1, \ldots, A_r)$ be a measurable partition of the sample space Θ. Let $K_t = \{l = 1, \ldots, L : \phi_l \in A_t\}$, $t = 1, \ldots, r$, where $r \leq L$. Assuming that G_0 is non-atomic, the ϕ_l's are distinct with

probability one, implying that any partition of $\{1, \ldots, L\}$ corresponds to some partition of Θ. Thus, as $\boldsymbol{\beta}_j^{(k)} \mid \alpha_j^{(k)}, \boldsymbol{\nu}_j^{(k,k)} \sim Dir\left(\alpha_j^{(k)}\left(\nu_{j1}^{(k,k)}, \ldots, \nu_{jL}^{(k,k)}\right)\right)$, from the properties of Dirichlet distribution, we have,

$$\left(G_j^{(k)\,L}(A_1), \ldots, G_j^{(k)\,L}(A_r)\right) = \left(\sum_{l \in K_1} \beta_{jl}^{(k)}, \ldots, \sum_{l \in K_r} \beta_{jl}^{(k)}\right)$$
$$\sim Dir\left(\alpha_j^{(k)} \sum_{l \in K_1} \nu_{jl}^{(k,k)}, \ldots, \alpha_j^{(k)} \sum_{l \in K_r} \nu_{jl}^{(k,k)}\right).$$

Thus,

$$G_j^{(k)\,L} \mid \alpha_j^{(k)}, H_j^{(k,k)\,L} \sim DP\left(\alpha_j^{(k)}, H_j^{(k,k)\,L}\right).$$

Similarly,

$$H_j^{(k,k)\,L} \mid \{\alpha_l^{(k-1)} : l \in an^{(k,1)}(j)\}, H_j^{(k,k-1)\,L} \sim DP\left(\sum_{l \in an^{(k,1)}(j)} \alpha_l^{(k-1)}, H_j^{(k,k-1)\,L}\right),$$
$$H_j^{(k,k-1)\,L} \mid \{\alpha_l^{(k-2)} : l \in an^{(k,2)}(j)\}, H_j^{(k,k-2)\,L} \sim DP\left(\sum_{l \in an^{(k,2)}(j)} \alpha_l^{(k-2)}, H_j^{(k,k-2)\,L}\right),$$
$$\vdots$$
$$H_j^{(k,2)\,L} \mid \{\alpha_l^{(1)} : l \in an^{(k,k-1)}(j)\}, G_1^{(0)\,L} \sim DP\left(\sum_{l \in an^{(k,k-1)}(j)} \alpha_l^{(1)}, G_1^{(0)\,L}\right).$$

By letting $L \to \infty$, the marginal distribution that this finite mixture model induces on the observations, $\boldsymbol{x}_j^{(k)} = (x_{j1}^{(k)}, x_{j2}^{(k)}, \ldots)$, approaches the proposed GDP mixture model. ■

Appendix D. Finite Mixture Model Approximation and Posterior Inference

The posterior inference of the proposed GDP mixture model is carried out using a blocked Gibbs sampler. For concreteness, we will present the finite mixture model approximation of the GDP for our motivating example and posterior inference based on this approximation. In our motivating application, we have 8 experimental groups. Each group corresponds to a combination of treatment, diet, and genotype; see Table 3 where we use binary indicators to denote the genotype, the two levels of diet, and the two treatment regimes. The design of the experiments naturally introduces dependencies among the experimental groups, which are represented by the DAG in Figure 12, where group 1 is the root node, groups 2-4 are the layer-1 nodes, groups 5-7 are the layer-2 nodes, and group 8 is the layer-3 node. For ease of notation, instead of using $G_1^{(0)}$ and $\alpha_1^{(0)}$ to denote the random measure and the concentration parameter of the root node, we use simply G_1 and α_1 instead; similarly for all the other nodes.

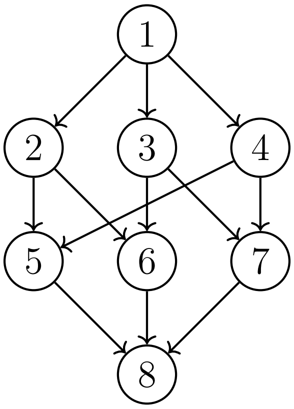

Figure 12: The DAG of experimental groups.

Group	Diet	Treatment	Genotype
1	0	0	0
2	1	0	0
3	0	1	0
4	0	0	1
5	1	0	1
6	1	1	0
7	0	1	1
8	1	1	1

Table 3: Each experimental group corresponds to a combination of diet, treatment, and genotype. Diet = 1 corresponds to high-fat diet and 0 corresponds to normal diet, Treatment = 1 corresponds to AdipoRon and 0 corresponds to no therapy, Genotype = 1 corresponds to Apc knock-out and 0 corresponds to wild type.

Recall that from the main text, the finite truncation of the infinite mixture model representation is given by,

$$\begin{aligned}
\boldsymbol{\beta}_1^{(0)} \mid \alpha_1^{(0)} &\sim Dir\left(\alpha_1^{(0)}/L, \ldots, \alpha_1^{(0)}/L\right), \\
\boldsymbol{\nu}_j^{(k,2)} \mid \{\alpha_l^{(1)} : l \in an^{(k,k-1)}(j)\}, \boldsymbol{\beta}_1^{(0)} &\sim Dir\left(\sum_{l \in an^{(k,k-1)}(j)} \alpha_l^{(1)} \left(\beta_{11}^{(0)}, \ldots, \beta_{1L}^{(0)}\right)\right), \\
&\vdots \\
\boldsymbol{\nu}_j^{(k,k)} \mid \{\alpha_l^{(k,k-1)} : l \in an^{(k,1)}(j)\}, \boldsymbol{\nu}_j^{(k-1)} &\sim Dir\left(\sum_{l \in an^{(k,1)}(j)} \alpha_l^{(k-1)} \left(\nu_{j1}^{(k,k-1)}, \ldots, \nu_{jL}^{(k,k-1)}\right)\right), \quad (29) \\
\boldsymbol{\beta}_j^{(k)} \mid \alpha_j^{(k)}, \boldsymbol{\nu}_j^{(k,k)} &\sim Dir\left(\alpha_j^{(k)} \left(\nu_{j1}^{(k,k)}, \ldots, \nu_{jL}^{(k,k)}\right)\right), \\
\phi_l \mid G_0 &\sim G_0, \\
z_{ji}^{(k)} \mid \boldsymbol{\beta}_j^{(k)} &\sim \boldsymbol{\beta}_j^{(k)}, \\
x_{ji}^{(k)} \mid z_{ji}^{(k)}, (\phi_l)_{l=1}^L &\sim F\left(\phi_{z_{ji}^{(k)}}\right).
\end{aligned}$$

Using the simplified notations for the group-specific random measures and concentration parameter, from the finite truncation of the infinite mixture model representation from Eq. (29), we have, for this motivating problem,

$$
\begin{aligned}
\alpha_1 \mid \alpha_0 &\sim Gamma(\alpha_0, 1), & \boldsymbol{\beta}_1 \mid \alpha_1 &\sim Dir(\alpha_1/L, \ldots, \alpha_1/L),\\
\alpha_2 \mid \alpha_1 &\sim Gamma(\alpha_1, 1), & \boldsymbol{\beta}_2 \mid \alpha_2, \boldsymbol{\beta}_1 &\sim Dir(\alpha_2 \boldsymbol{\beta}_1),\\
\alpha_3 \mid \alpha_1 &\sim Gamma(\alpha_1, 1), & \boldsymbol{\beta}_3 \mid \alpha_3, \boldsymbol{\beta}_1 &\sim Dir(\alpha_3 \boldsymbol{\beta}_1),\\
\alpha_4 \mid \alpha_1 &\sim Gamma(\alpha_1, 1), & \boldsymbol{\beta}_4 \mid \alpha_4, \boldsymbol{\beta}_1 &\sim Dir(\alpha_4 \boldsymbol{\beta}_1),\\
\alpha_5 \mid \alpha_2, \alpha_4 &\sim Gamma(\alpha_2 + \alpha_4, 1), & \boldsymbol{\beta}_5 \mid \alpha_5, \boldsymbol{\nu}_1 &\sim Dir(\alpha_5 \boldsymbol{\nu}_1),\\
& & \boldsymbol{\nu}_1 \mid \alpha_2, \alpha_4, \boldsymbol{\beta}_1 &\sim Dir\left((\alpha_2 + \alpha_4)\, \boldsymbol{\beta}_1\right),\\
\alpha_6 \mid \alpha_2, \alpha_3 &\sim Gamma(\alpha_2 + \alpha_3, 1), & \boldsymbol{\beta}_6 \mid \alpha_6, \boldsymbol{\nu}_2 &\sim Dir(\alpha_6 \boldsymbol{\nu}_2),\\
& & \boldsymbol{\nu}_2 \mid \alpha_2, \alpha_3, \boldsymbol{\beta}_1 &\sim Dir\left((\alpha_2 + \alpha_3)\, \boldsymbol{\beta}_1\right),\\
\alpha_7 \mid \alpha_3, \alpha_4 &\sim Gamma(\alpha_3 + \alpha_4, 1), & \boldsymbol{\beta}_7 \mid \alpha_7, \boldsymbol{\nu}_3 &\sim Dir(\alpha_7 \boldsymbol{\nu}_3),\\
& & \boldsymbol{\nu}_3 \mid \alpha_3, \alpha_4, \boldsymbol{\beta}_1 &\sim Dir\left((\alpha_3 + \alpha_4)\, \boldsymbol{\beta}_1\right),\\
\alpha_8 \mid \alpha_5, \alpha_6, \alpha_7 &\sim Gamma(\alpha_5 + \alpha_6 + \alpha_7, 1), & \boldsymbol{\beta}_8 \mid \boldsymbol{\nu}_4, \alpha_8, &\sim Dir(\alpha_8 \boldsymbol{\nu}_4),\\
& & \boldsymbol{\nu}_4 \mid \alpha_5, \alpha_6, \alpha_7, \boldsymbol{\eta} &\sim Dir\left((\alpha_5 + \alpha_6 + \alpha_7)\, \boldsymbol{\eta}\right),\\
& & \boldsymbol{\eta} \mid \alpha_2, \alpha_3, \alpha_4, \boldsymbol{\beta}_1 &\sim Dir\left(2(\alpha_2 + \alpha_3 + \alpha_4)\, \boldsymbol{\beta}_1\right),\\
z_{ji} \mid \boldsymbol{\beta}_j &\overset{ind}{\sim} Cat(1 : L, \boldsymbol{\beta}_j),\\
x_{ji} \mid z_{ji}, (\phi_l)_{l=1}^{L} &\overset{ind}{\sim} F(\phi_{z_{ji}}), & i = 1, \ldots, n_j,\ \ j &= 1, \ldots, 8, \qquad (30)
\end{aligned}
$$

where $\boldsymbol{\beta}_1 = (\beta_{11}, \ldots, \beta_{1L})$, $\boldsymbol{\nu}_1 = (\nu_{11}, \ldots, \nu_{1L})$, $\boldsymbol{\nu}_2 = (\nu_{21}, \ldots, \nu_{2L})$, $\boldsymbol{\nu}_3 = (\nu_{31}, \ldots, \nu_{3L})$, $\boldsymbol{\nu}_4 = (\nu_{41}, \ldots, \nu_{4L})$, and $\boldsymbol{\eta} = (\eta_1, \ldots, \eta_L)$. With the above distributional structure, Gibbs sampling is straightforward. We use $\pi(.)$ and $\pi(. \mid -)$ to denote the prior distribution and the conditional distribution, respectively, of the parameter specified in the argument. The full conditional distribution for the atoms is given by,

$$
\pi(\{\phi_l\}_{l=1}^{L} \mid -) \propto \prod_{l=1}^{L} \left[\left\{ \prod_{j=1}^{8} \prod_{i=1}^{n_j} F(x_{ji} \mid \phi_l)^{\mathbb{1}(z_{ji}=l)} \right\} \pi(\phi_l) \right]. \tag{31}
$$

The full conditional distributions for the latent cluster labels are given by,

$$
P(z_{ji} = l \mid -) \propto \beta_{jl} F(x_{ji} \mid \phi_l), \quad l = 1, \ldots, L, \quad i = 1, \ldots, n_j \quad j = 1, \ldots, 8. \tag{32}
$$

The full conditional distribution for the stick-breaking weights is given by,

$$
\begin{aligned}
\pi(\boldsymbol{\beta}_1 \mid -) \propto \frac{\prod_{l=1}^{L} \beta_{1l}^{m_{1l} + \frac{\alpha_1}{L}} \left\{ \beta_{2l}^{\alpha_2} \beta_{3l}^{\alpha_3} \beta_{4l}^{\alpha_4} \nu_{1l}^{\alpha_2+\alpha_4} \nu_{2l}^{\alpha_2+\alpha_3} \nu_{3l}^{\alpha_3+\alpha_4} \eta_l^{2(\alpha_2+\alpha_3+\alpha_4)} \right\}^{\beta_{1l}}}{\prod_{l=1}^{L} \left\{ \Gamma\left((\alpha_2+\alpha_4)\beta_{1l}\right) \Gamma\left((\alpha_2+\alpha_3)\beta_{1l}\right) \Gamma\left((\alpha_3+\alpha_4)\beta_{1l}\right) \Gamma\left(2(\alpha_2+\alpha_3+\alpha_4)\beta_{1l}\right) \right\}} \\
\times \frac{1}{\prod_{l=1}^{L} \left\{ \Gamma\left(\alpha_2 \beta_{1l}\right) \Gamma\left(\alpha_3 \beta_{1l}\right) \Gamma\left(\alpha_4 \beta_{1l}\right) \right\}},
\end{aligned} \tag{33}
$$

where $m_{1l} = \sum_{i=1}^{n_1} \mathbb{1}(z_{1i} = l)$, $l = 1, \ldots, L$. The full conditionals for $\boldsymbol{\beta}_j$, $j = 2, \ldots, 8$, are in closed form,

$$
\pi(\boldsymbol{\beta}_j \mid -) \sim Dir(\boldsymbol{m}_j + \alpha_j \boldsymbol{\beta}_1), \quad \text{where } \boldsymbol{m}_j = (m_{j1}, \ldots, m_{jL}) \text{ and } m_{jl} = \sum_{i=1}^{n_j} \mathbb{1}(z_{ji} = l),\ l = 1, \ldots, L. \tag{34}
$$

By letting $\boldsymbol{B}(\boldsymbol{a})$ to denote the multivariate beta function, i.e., for a L-dimensional vector $\boldsymbol{a} = (a_1, \ldots, a_L)$ with $a_i > 0$, we have,

$$
\boldsymbol{B}(\boldsymbol{a}) = \frac{\prod_{l=1}^{L} \Gamma(a_l)}{\Gamma(\sum_{l=1}^{L} a_l)},
$$

where $\Gamma(\cdot)$ is the gamma function. Then the full-conditional distribution of the hidden weights are given by,

$$\pi(\boldsymbol{\nu}_1 \mid -) \propto \frac{1}{\boldsymbol{B}(\alpha_5 \boldsymbol{\nu}_1)} \prod_{l=1}^{L} \left\{ \beta_{5l}^{\alpha_5 \nu_{1l}} \nu_{1l}^{(\alpha_2+\alpha_4)\beta_{1l}-1} \right\}, \tag{35}$$

$$\pi(\boldsymbol{\nu}_2 \mid -) \propto \frac{1}{\boldsymbol{B}(\alpha_6 \boldsymbol{\nu}_2)} \prod_{l=1}^{L} \left\{ \beta_{6l}^{\alpha_6 \nu_{2l}} \nu_{2l}^{(\alpha_2+\alpha_3)\beta_{1l}-1} \right\}, \tag{36}$$

$$\pi(\boldsymbol{\nu}_3 \mid -) \propto \frac{1}{\boldsymbol{B}(\alpha_7 \boldsymbol{\nu}_3)} \prod_{l=1}^{L} \left\{ \beta_{7l}^{\alpha_7 \nu_{3l}} \nu_{3l}^{(\alpha_3+\alpha_4)\beta_{1l}-1} \right\}, \tag{37}$$

$$\pi(\boldsymbol{\nu}_4 \mid -) \propto \frac{1}{\boldsymbol{B}(\alpha_8 \boldsymbol{\nu}_4)} \prod_{l=1}^{L} \left\{ \beta_{4l}^{\alpha_8 \nu_{4l}} \nu_{4l}^{(\alpha_5+\alpha_6+\alpha_7)\eta_l-1} \right\}, \tag{38}$$

$$\pi(\boldsymbol{\eta} \mid -) \propto \frac{1}{\boldsymbol{B}((\alpha_5+\alpha_6+\alpha_7)\boldsymbol{\eta})} \prod_{l=1}^{L} \left\{ \eta_l^{2(\alpha_2+\alpha_3+\alpha_4)\beta_{1l}-1} \nu_{4l}^{(\alpha_5+\alpha_6+\alpha_7)\eta_l} \right\}. \tag{39}$$

The full conditionals for the concentration parameters are given by,

$$\pi(\alpha_1 \mid -) \propto \frac{e^{-\alpha_1} \alpha_1^{\alpha_0-1} \alpha_2^{\alpha_1} \alpha_3^{\alpha_1} \alpha_4^{\alpha_1}}{\{\Gamma(\alpha)\}^3 \boldsymbol{B}((\alpha_1/L, \ldots, \alpha_1/L)} \prod_{l=1}^{L} \beta_{1l}^{\frac{\alpha_1}{L}} \tag{40}$$

$$\pi(\alpha_2 \mid -) \propto \frac{e^{-\alpha_2} \alpha_2^{\alpha_1-1} \alpha_5^{\alpha_2} \alpha_6^{\alpha_2} \left[\prod_{l=1}^{L} \left\{ \beta_{2l}^{\beta_{1l}} \nu_{1l}^{\beta_{1l}} \nu_{2l}^{\beta_{1l}} \eta_l^{2\beta_{1l}} \right\}^{\alpha_2} \right] \Gamma(\alpha_2) \Gamma(2(\alpha_2+\alpha_3+\alpha_4))}{\prod_{l=1}^{L} \{ \Gamma(\alpha_2 \beta_{1l}) \Gamma((\alpha_2+\alpha_4)\beta_{1l}) \Gamma((\alpha_2+\alpha_3)\beta_{1l}) \Gamma(2(\alpha_2+\alpha_3+\alpha_4)\beta_{1l}) \}}, \tag{41}$$

$$\pi(\alpha_3 \mid -) \propto \frac{e^{-\alpha_3} \alpha_3^{\alpha_1-1} \alpha_6^{\alpha_3} \alpha_7^{\alpha_3} \left[\prod_{l=1}^{L} \left\{ \beta_{3l}^{\beta_{1l}} \nu_{2l}^{\beta_{1l}} \nu_{3l}^{\beta_{1l}} \eta_l^{2\beta_{1l}} \right\}^{\alpha_3} \right] \Gamma(\alpha_3) \Gamma(2(\alpha_2+\alpha_3+\alpha_4))}{\prod_{l=1}^{L} \{ \Gamma(\alpha_3 \beta_{1l}) \Gamma((\alpha_2+\alpha_3)\beta_{1l}) \Gamma((\alpha_3+\alpha_4)\beta_{1l}) \Gamma(2(\alpha_2+\alpha_3+\alpha_4)\beta_{1l}) \}}, \tag{42}$$

$$\pi(\alpha_4 \mid -) \propto \frac{e^{-\alpha_4} \alpha_4^{\alpha_1-1} \alpha_5^{\alpha_4} \alpha_7^{\alpha_4} \left[\prod_{l=1}^{L} \left\{ \beta_{4l}^{\beta_{1l}} \nu_{1l}^{\beta_{1l}} \nu_{3l}^{\beta_{1l}} \eta_l^{2\beta_{1l}} \right\}^{\alpha_4} \right] \Gamma(\alpha_4) \Gamma(2(\alpha_2+\alpha_3+\alpha_4))}{\prod_{l=1}^{L} \{ \Gamma(\alpha_4 \beta_{1l}) \Gamma((\alpha_2+\alpha_4)\beta_{1l}) \Gamma((\alpha_3+\alpha_4)\beta_{1l}) \Gamma(2(\alpha_2+\alpha_3+\alpha_4)\beta_{1l}) \}}, \tag{43}$$

$$\pi(\alpha_5 \mid -) \propto \frac{e^{-\alpha_5} \alpha_5^{\alpha_2+\alpha_4-1} \alpha_8^{\alpha_5} \left[\prod_{l=1}^{L} \{ \beta_{5l}^{\nu_{1l}} \nu_{4l}^{\eta_l} \}^{\alpha_5} \right] \Gamma(\alpha_5)}{\prod_{l=1}^{L} \{ \Gamma(\alpha_5 \nu_{1l}) \Gamma((\alpha_5+\alpha_6+\alpha_7)\nu_l) \}}, \tag{44}$$

$$\pi(\alpha_6 \mid -) \propto \frac{e^{-\alpha_6} \alpha_6^{\alpha_2+\alpha_3-1} \alpha_8^{\alpha_6} \left[\prod_{l=1}^{L} \{ \beta_{6l}^{\nu_{2l}} \nu_{4l}^{\eta_l} \}^{\alpha_6} \right] \Gamma(\alpha_6)}{\prod_{l=1}^{L} \{ \Gamma(\alpha_6 \nu_{2l}) \Gamma((\alpha_5+\alpha_6+\alpha_7)\nu_l) \}}, \tag{45}$$

$$\pi(\alpha_7 \mid -) \propto \frac{e^{-\alpha_7} \alpha_7^{\alpha_3+\alpha_4-1} \alpha_8^{\alpha_7} \left[\prod_{l=1}^{L} \{ \beta_{7l}^{\nu_{3l}} \nu_{4l}^{\eta_l} \}^{\alpha_7} \right] \Gamma(\alpha_7)}{\prod_{l=1}^{L} \{ \Gamma(\alpha_7 \nu_{3l}) \Gamma((\alpha_5+\alpha_6+\alpha_7)\nu_l) \}}, \tag{46}$$

$$\pi(\alpha_8 \mid -) \propto \frac{e^{-\alpha_8} \alpha_8^{\alpha_5+\alpha_6+\alpha_7-1} \left[\prod_{l=1}^{L} \beta_{8l}^{\alpha_8 \nu_{4l}} \right] \Gamma(\alpha_8)}{\prod_{l=1}^{L} \Gamma(\alpha_8 \nu_{4l})}. \tag{47}$$

Note that the full conditionals of α_j, $j = 1, \ldots, 8$, $\boldsymbol{\beta}_1, \boldsymbol{\nu}_1, \boldsymbol{\nu}_2, \boldsymbol{\nu}_3, \boldsymbol{\nu}_4$, and $\boldsymbol{\eta}$ are not standard distributions that have direct samplers. We adopt a Metropolis-within-Gibbs strategy to sample from their corresponding full conditional distributions. Since α_j's are real-valued, sampling using a Metropolis step is straightforward. However, the main bottleneck in sampling are the weights $\boldsymbol{\beta}_1, \boldsymbol{\nu}_1, \boldsymbol{\nu}_2, \boldsymbol{\nu}_3, \boldsymbol{\nu}_4$ and $\boldsymbol{\eta}$, which have a complex structure on the simplex. To mitigate this problem, we use the SALTSampler (Director et al., 2017) for which the implementation is publicly available as an R package.

Appendix E. Simulation details

Our simulations are designed to mimick the motivating application where we have 8 experimental groups. See Table 3 for our experimental design represented in terms of binary indicators denoting the levels of diet, treatment, and genotype. The corresponding DAG is given in Figure 12.

For our simulation study, we generated data within each of the 8 groups from a four-component mixture of bivariate Gaussian distributions with different covariance matrices for each group. Taking $\alpha_0 = 5$, we drew the concentration parameters for the different groups α_j's, the mixture model weights, $\boldsymbol{\beta}_j$'s, $\boldsymbol{\nu}_j$'s, and $\boldsymbol{\eta}_j$, and the true cluster indicators z_{ji}'s for each of the different groups using (30). Given the cluster indicators, the data were generated from the Gaussian distribution with the true cluster-specific means ϕ_l's given in Table 4 and the group-specific covariance matrices given in Table 5. Note that within each group, the same covariance matrix was used for all clusters.

Cluster	Mean
1	(-2, -5)
2	(0, 0)
3	(-3, 3)
4	(3, -3)

Table 4: True cluster-specific means.

Group	Covariance
1	$\begin{bmatrix} 0.8 & 0.3 \\ 0.3 & 0.8 \end{bmatrix}$
2	$\begin{bmatrix} 0.85 & 0.25 \\ 0.25 & 0.85 \end{bmatrix}$
3	$\begin{bmatrix} 1 & 0.1 \\ 0.1 & 1 \end{bmatrix}$
4	$\begin{bmatrix} 0.8 & -0.1 \\ -0.1 & 0.8 \end{bmatrix}$
5	$\begin{bmatrix} 0.8 & -0.2 \\ -0.2 & 0.9 \end{bmatrix}$
6	$\begin{bmatrix} 0.8 & 0 \\ 0 & 0.8 \end{bmatrix}$
7	$\begin{bmatrix} 0.75 & 0.25 \\ 0.25 & 0.75 \end{bmatrix}$
8	$\begin{bmatrix} 1.1 & 0.1 \\ 0.1 & 1.1 \end{bmatrix}$

Table 5: True covariance matrices for different groups.

In our Gibbs sampler, the truncation level of the finite mixture model was set to $L = 10$, and the base measure for GDP, G_0, was specified as the normal-inverse-Wishart distribution, $\mathcal{NIW}(\mathbf{0}, 0.01, \mathbb{I}_2, 2)$. Upon the completion of the Gibbs sampler, the clusters were estimated by

using the least squares criterion (Dahl, 2006), and they were compared with the true cluster labels for evaluation. We considered a variety of sample sizes as well as a case with very imbalanced design, which are summarized in Table 6. In all cases, we ran 15,000 iterations of our Gibbs sampler and after discarding the first 5,000 samples as burn-in, we retained every 10th iteration of posterior samples.

Group	Sample sizes			
	small	moderate	large	unbalanced
1	40	80	150	350
2	30	70	160	30
3	30	70	180	40
4	35	75	170	45
5	25	83	155	25
6	30	88	175	25
7	25	92	185	35
8	30	88	145	35

Table 6: The sample sizes for the different groups that were used to simulate the data.

We presented the results of clustering for small sample sizes and unbalanced sample sizes in the main manuscript. Figure 13 shows the results of clustering for moderate and large sample sizes in each group.

We further considered the case, wherein the simulation scenario was difficult. We generated data within each of the 8 groups from a ten-component mixture of bivariate Gaussian distributions with different covariance matrices for each group. The choice of mixture model weights for the first fours groups are summarized in Table 7.

Group	Mixture weights $\boldsymbol{\beta}_j$
1	$(0.100, 0.100, 0.100, 0.100, 0.100, 0.100, 0.100, 0.100, 0.100, 0.100)^\top$
2	$(0.167, 0.167, 0.167, 0.167, 0.167, 0.056, 0.056, 0.056, 0.000, 0.000)^\top$
3	$(0.095, 0.095, 0.095, 0.000, 0.000, 0.143, 0.143, 0.143, 0.143, 0.143)^\top$
4	$(0.030, 0.030, 0.030, 0.182, 0.182, 0.182, 0.182, 0.182, 0.000, 0.000)^\top$

Table 7: True group-specific mixture model weights.

The mixture weights for all other groups were taken to be the mean of the mixture weights of their parent, e.g., the mixture weight for group 5 was the mean of the mixture weights of groups 2 and 4. The true cluster indicators z_{ji}'s for each of the different groups were drawn using (30) and the true mixture weights. Given the cluster indicators, the data were generated from the Gaussian distribution with the true cluster-specific means ϕ_l's given in Table 8 and the group-specific covariance matrices given in Table 5.

In our Gibbs sampler, the truncation level of the finite mixture model was set to $L = 20$, the hyperparameter α_0 was taken to be 1, and the base measure for GDP, G_0, was specified as the normal-inverse-Wishart distribution, $\mathcal{NIW}(\mathbf{0}, 0.01, \mathbb{I}_2, 2)$. Upon the completion of the Gibbs sampler, the clusters were estimated by using the least squares criterion (Dahl, 2006), and they were compared with the true cluster labels for evaluation. We again considered a variety of sample sizes as summarized in Table 6. In all cases, we ran 25,000 iterations of our Gibbs sampler and after discarding the first 15,000 samples as burn-in, considered thinning of the samples by a factor 10. The clustering results are shown in Figure 14. Clearly, GDP was able to identify the overlapping clusters

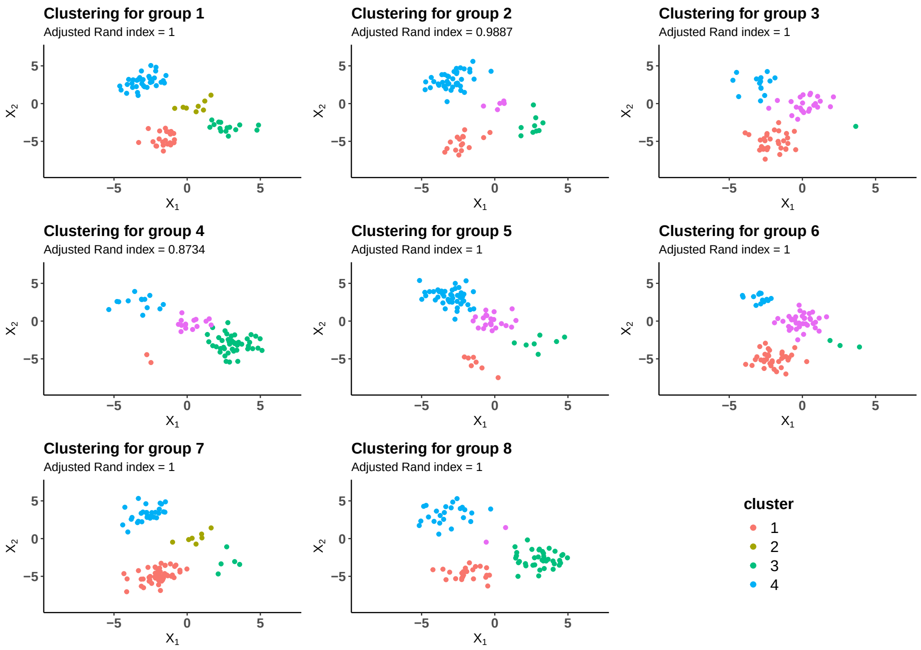

(a) Moderate sample size in each group

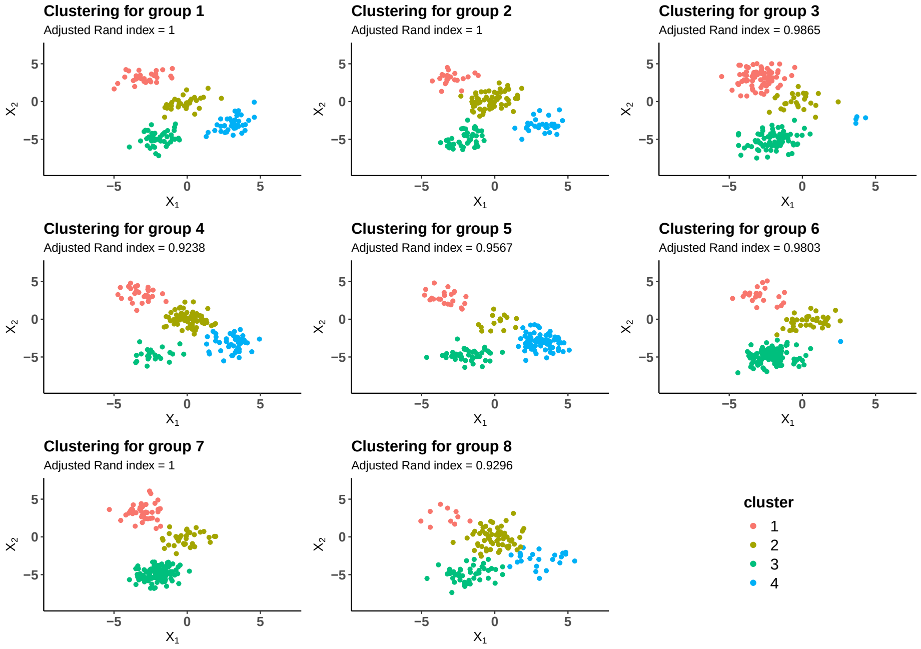

(b) Large sample size in each group

Figure 13: Clustering performance of GDP for additional sample sizes. The colors indicate the estimated clusters by GDP. Adjusted Rand index is reported at the top of each panel.

Cluster	Mean
1	(-2.5, 0)
2	(0, 0)
3	(2.5, 0)
4	(2.5, -2.5)
5	(-3, -3)
6	(2, 2)
7	(-2, 5)
8	(5, 8)
9	(-5, -8)
10	(8, -8)

Table 8: True cluster-specific means.

within each group and link them across groups for all simulation scenarios with reasonable accuracy as measured by the adjusted Rand indices (Hubert and Arabie, 1985) for each group (shown in the plots).

In the main manuscript, we reported the boxplot of Adjusted Rand indices for 50 replicates. Further investigation regarding the choice of α_0 revealed no significant impact in clustering performance. Figure 15 shows that boxplot of Adjusted Rand indices for 50 replicates, comparing GDP, HDP, and k-means with α_0 taken to be 6. In all situations, GDP uniformly out-performed the other two methods.

Appendix F. Additional simulations

In the main manuscript, we presented simulations to mimick the motivating application where we have 8 experimental groups. However, there are several motivating applications of the proposed GDP. One such example is time-series data. One might be interested in clustering stocks based on daily prices for each year. Each calendar year is then a group. The groups naturally have time dependence (i.e., one does not expect the clustering of stocks to change dramatically in consecutive years), which may be represented by an autoregressive (AR) model. AR model is one type of DAG model. Particularly, for an AR model with lag 1, the time dependencies can be represented by a simple DAG (see Figure 16), which may be analyzed by the GDP. With this specific DAG (chain DAG), the GDP is given by,

$$G_1 \mid \alpha_1, G_0 \sim DP\left(\alpha_1, G_0\right), \tag{48}$$

$$G_t \mid \alpha_t, G_{t-1} \sim DP\left(\alpha_t, G_{t-1}\right), \quad t = 2, \ldots, T, \tag{49}$$

where T denotes the total number of observed time points. Let x_{ti} denote the observation i from time point t and θ_{ti} denote the parameter specifying the mixture component associated with the corresponding observation. Let $F(\theta_{ti})$ denote the distribution of x_{ti} given θ_{ti} and G_t denote a prior distribution for θ_{ti}. The group-specific mixture model is given by,

$$\begin{aligned} \theta_{ti} \mid G_t &\overset{ind}{\sim} G_t, \\ x_{ti} \mid \theta_{ti} &\overset{ind}{\sim} F(\theta_{ti}), \end{aligned} \tag{50}$$

where G_t follows (48) and (49). The observations $x_{t1}, x_{t2}, \ldots, x_{tn_t}$ are exchangeable at each observed time point t but the groups (formed at the different time points) are not exchangeable due to the time-dependency between the groups. The corresponding GDP mixture model can be derived as the

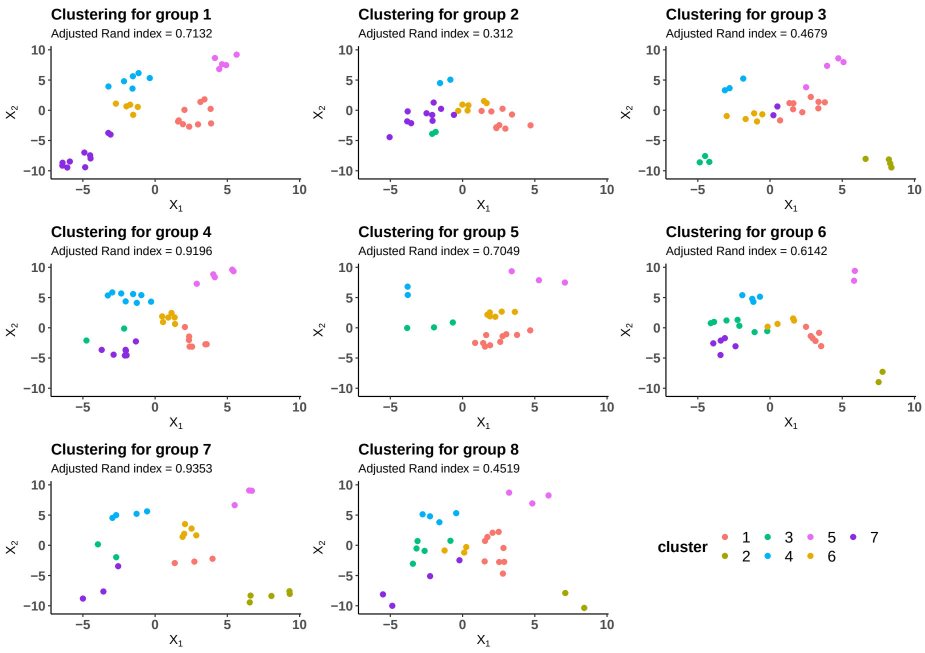

(a) Small sample size in each group

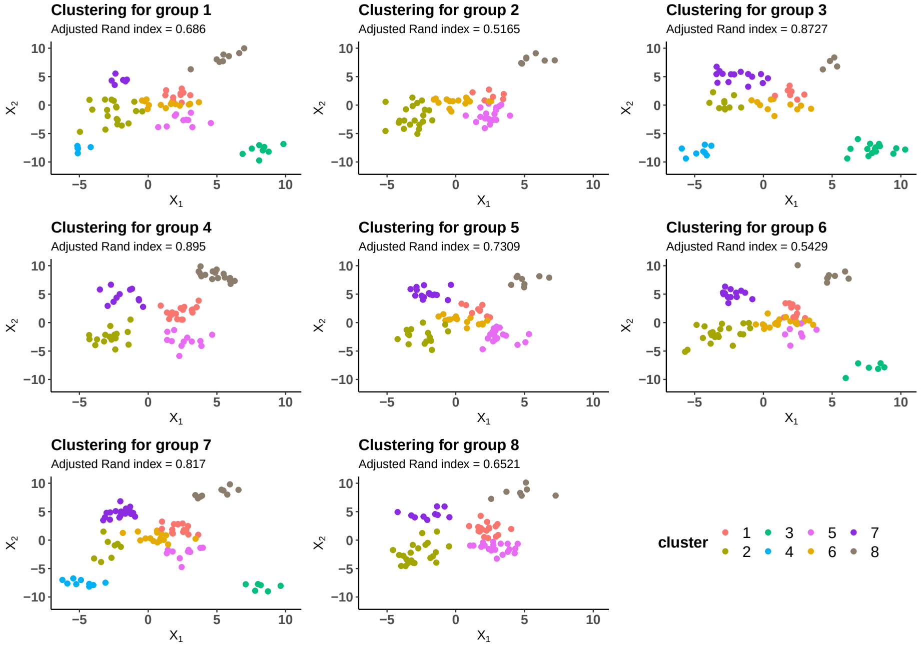

(b) Moderate sample size in each group

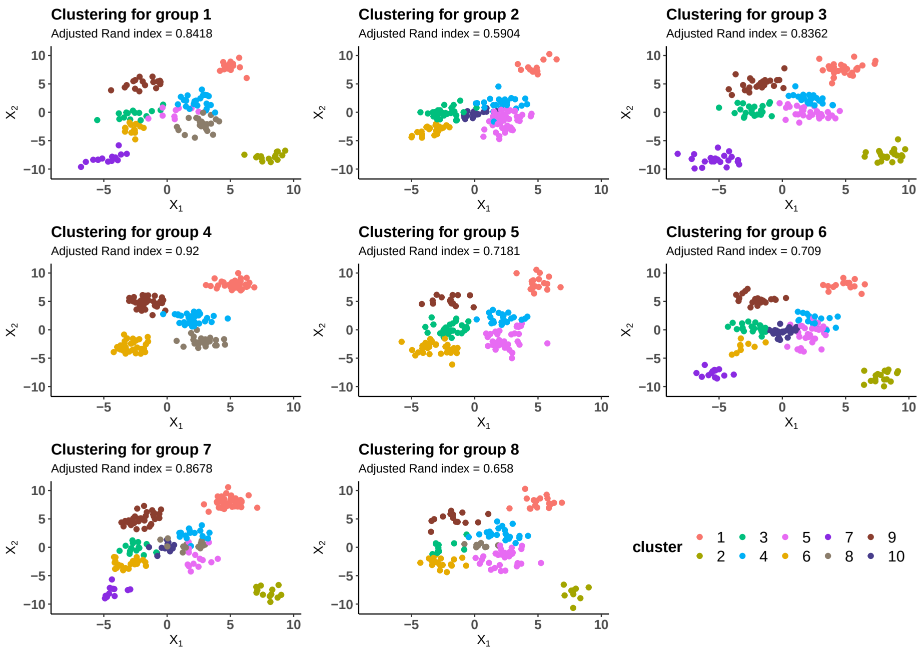

(c) Large sample size in each group

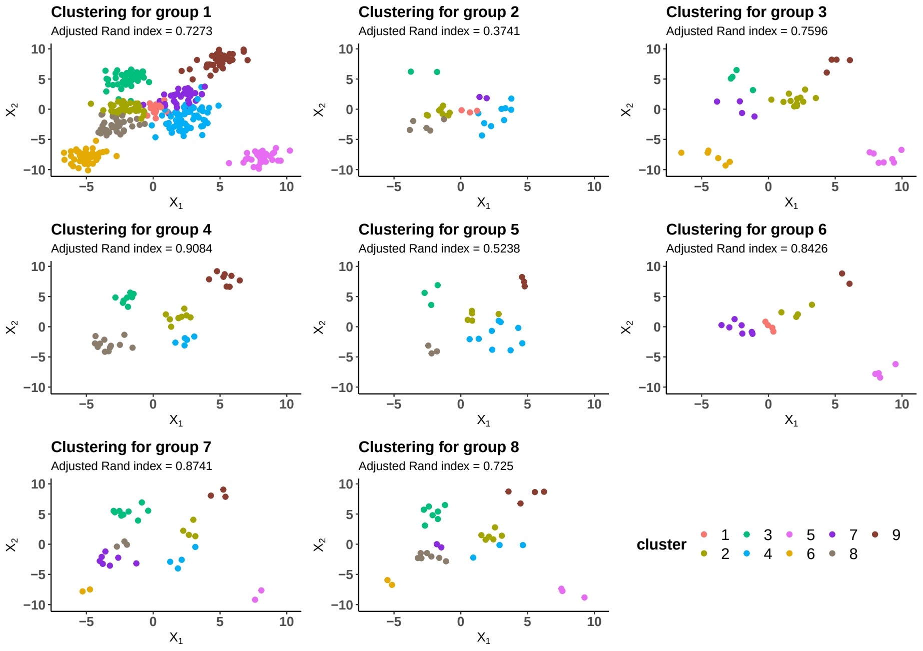

(d) Unbalanced sample size in each group

Figure 14: Clustering performance of GDP for various sample sizes and difficult simulation scenario. The colors indicate the estimated clusters by GDP. Adjusted Rand index is reported at the top of each panel.

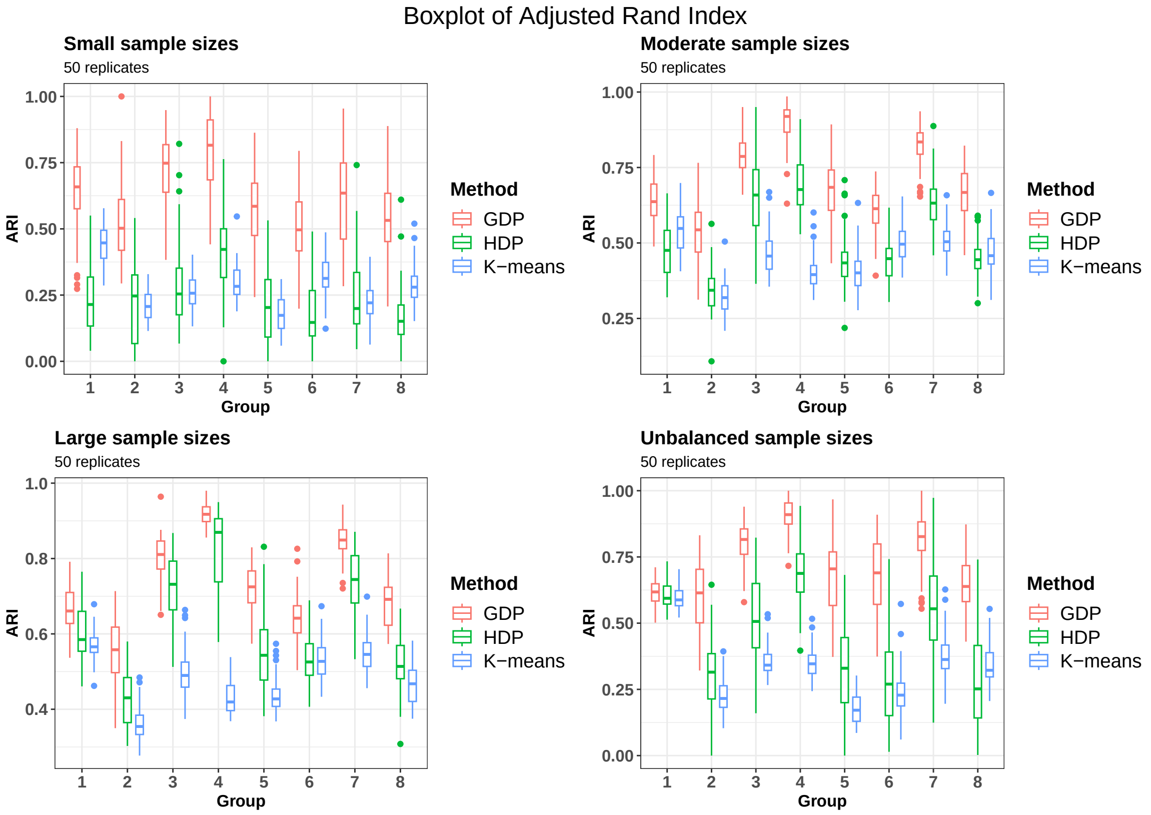

Figure 15: The boxplots of the Adjusted Rand indices for GDP, HDP, and k-means for all sample sizes. In all simulations α_0 was taken to be 6.

infinite limit of a finite mixture model. Let us denote the mixture component associated with the observation x_{ti} from time point t, by z_{ti}. Suppose $\boldsymbol{\beta}_1$ is the vector of mixing weights for the root node (corresponding to time point $t = 1$). Denoting by $\boldsymbol{\beta}_t$ the mixing weights of node t (corresponding to time point t), we consider a finite mixture version of the proposed GDP,

$$\begin{aligned}
\alpha_1 \mid \alpha_0 &\sim Gamma(\alpha_0, 1), \\
\boldsymbol{\beta}_1 \mid \alpha_1 &\sim Dir\left(\alpha_1/L, \ldots, \alpha_1/L\right), \\
\alpha_t \mid \alpha_{t-1} &\sim Gamma(\alpha_{t-1}, 1), \\
\boldsymbol{\beta}_t \mid \alpha_t, \boldsymbol{\beta}_{t-1} &\sim Dir\left(\alpha_t \left(\beta_{t1}, \ldots, \beta_{tL}\right)\right), \\
\phi_l \mid G_0 &\sim G_0, \\
z_{ti} \mid \boldsymbol{\beta}_t &\sim \boldsymbol{\beta}_t, \\
x_{ti} \mid z_{ti}, (\phi_l)_{l=1}^{L} &\sim F\left(\phi_{z_{ti}}\right), \quad i = 1, \ldots, n_t.
\end{aligned} \tag{51}$$

We considered simple simulation examples, where we analyzed time-dependent observations (Figure 16) for $T = 10, 20$, and 50 time points. We generated data within each of the T time points (groups) from a five-component mixture of bivariate Gaussian distributions. Taking $\alpha_0 = 15$, we drew the concentration parameters for the different groups α_t's, the mixture model weights, $\boldsymbol{\beta}_t$'s, and the true cluster indicators z_{ti}'s for each of the different groups using (51). Given the cluster indicators, the data were generated from the Gaussian distribution with the true cluster-specific means ϕ_l's given in Table 9 and the same covariance matrix $\begin{bmatrix} 0.5 & 0.1 \\ 0.1 & 0.5 \end{bmatrix}$ across clusters, which was assumed to be known for simplicity. Furthermore, we considered 100 observations at each time point t, i.e., $n_t = 100$, $t = 1, \ldots, T$. In our Gibbs sampler, the truncation level of the finite mixture model was set to $L = 10$, and the base measure for GDP, G_0, was specified as the normal distribution, $\mathcal{N}(\mathbf{0}, 0.01^{-1}\mathbb{I}_2)$. We ran our MCMC for $50,000$ iterations, discarded the first $35,000$ iterations as burn-in, and retained every 15th posterior sample. Upon the completion of the Gibbs sampler, the clusters were estimated by using the least squares criterion (Dahl, 2006), and they were compared with the true cluster labels for evaluation. Figures 17 - 19 show the clustering plots for $T = 10, 20$, and 50 time points respectively. Clearly, our model was able to identify the clusters within each group and link them across groups (time points) with good accuracy as measured by adjusted Rand indices (ARI) for each group (shown in the plots). Furthermore, the traceplots of the log-likelihood showed no lack of convergence (Figure 20). Additionally, we considered 50 independent replications to investigate the runtime of our MCMC for varying number of nodes, T. Figure 21 shows that the runtime is approximately linear in the number of nodes T, for fixed truncation level L of the GDP. We further compared the clustering performance for the time-dependent grouped data using HDP. Figures 22 - 24 show the corresponding clustering plots corresponding to $T = 10, 20$, and 50 time points respectively. As before, HDP fails to capture meaningful clusters across the non-exchangeable groups, as indicated by the low ARI (shown in the plots).

Cluster	Mean
1	(-2, -5)
2	(0, 0)
3	(-3, 3)
4	(3, -3)
5	(8, 5)

Table 9: True cluster-specific means.

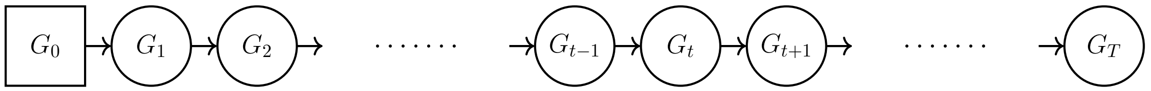

Figure 16: The DAG denoting time-dependency.

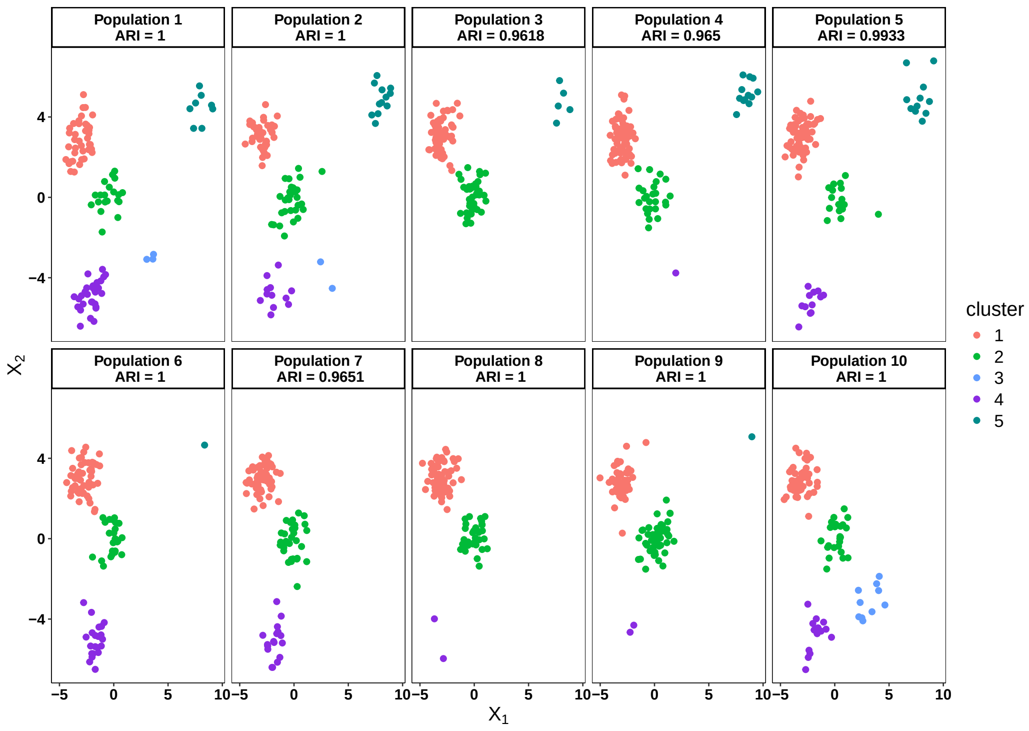

Figure 17: Clustering performance of time-dependent GDP for $T = 10$ time points. Population t refers to the observed group at time point t. The colors indicate the estimated clusters by GDP. Adjusted Rand index is reported at the top of each panel.

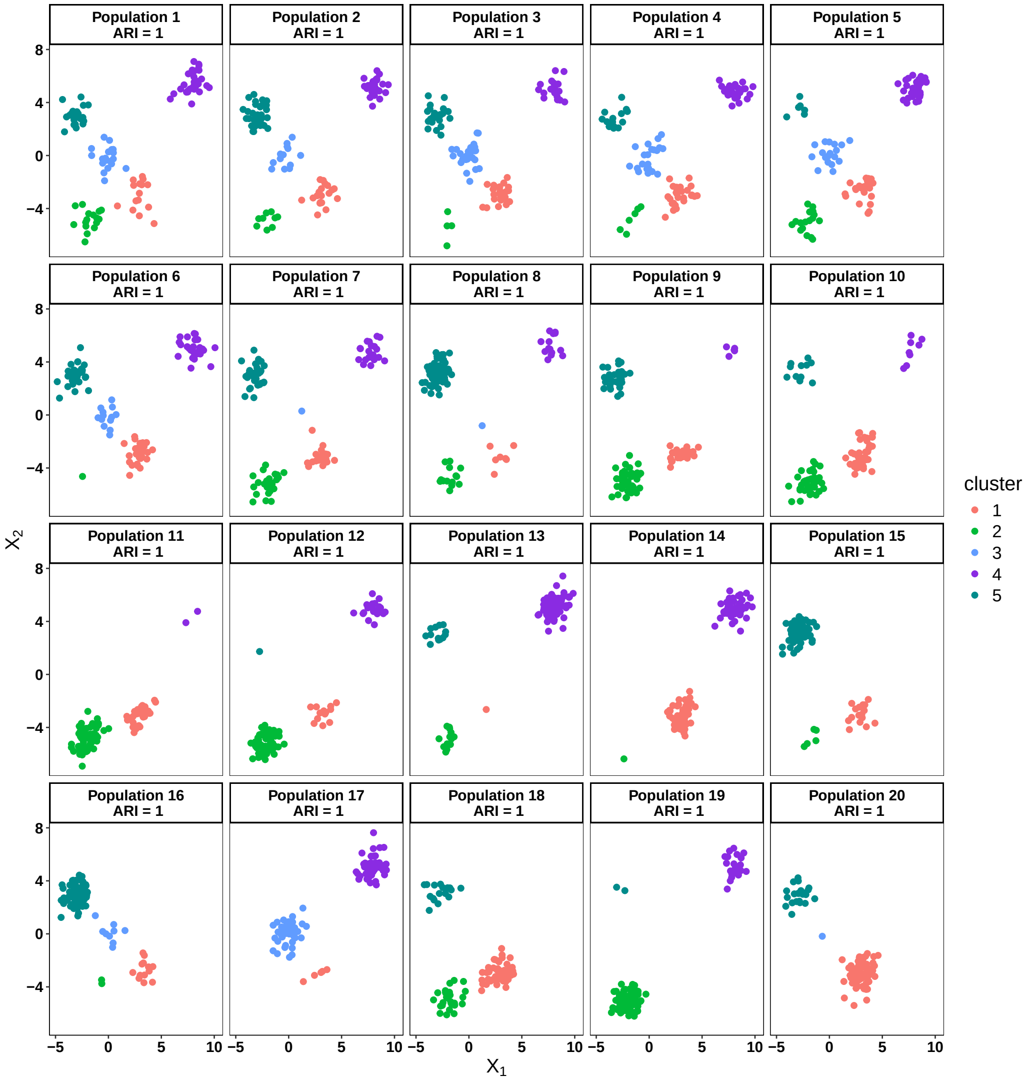

Figure 18: Clustering performance of time-dependent GDP for $T = 20$ time points. Population t refers to the observed group at time point t. The colors indicate the estimated clusters by GDP. Adjusted Rand index is reported at the top of each panel.

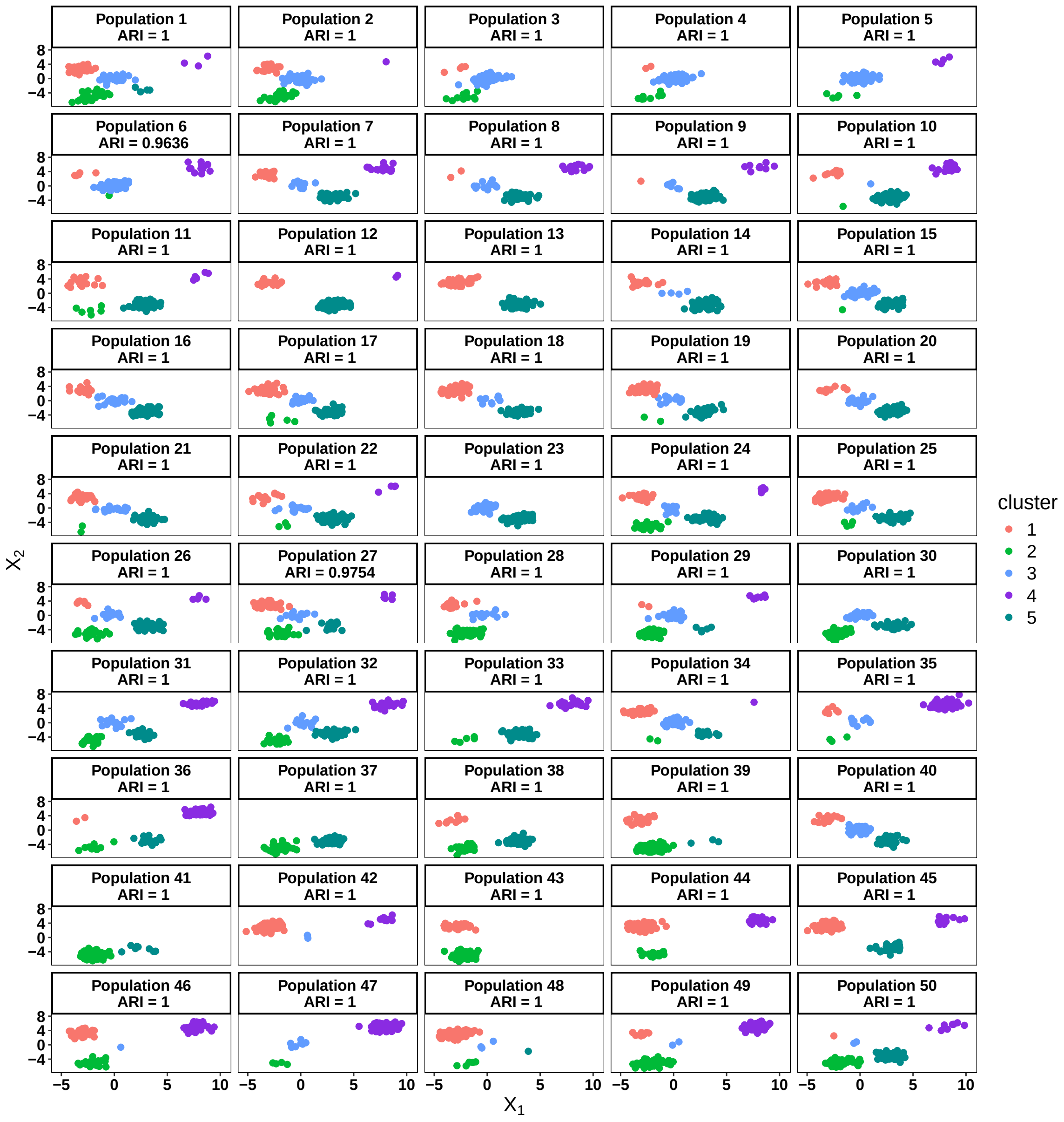

Figure 19: Clustering performance of time-dependent GDP for $T = 50$ time points. Population t refers to the observed group at time point t. The colors indicate the estimated clusters by GDP. Adjusted Rand index is reported at the top of each panel.

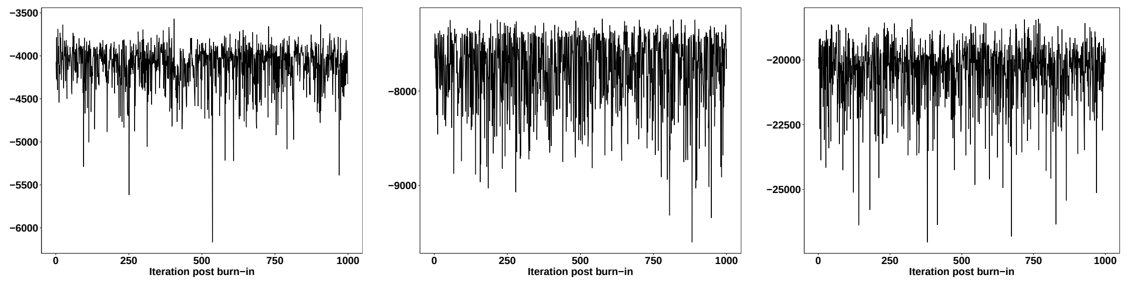

(a) $T = 10$ time points. (b) $T = 20$ time points. (c) $T = 50$ time points.

Figure 20: Traceplot of log-likelihood post burn-in and thinning for the varying number of nodes (T) in the chain DAG.

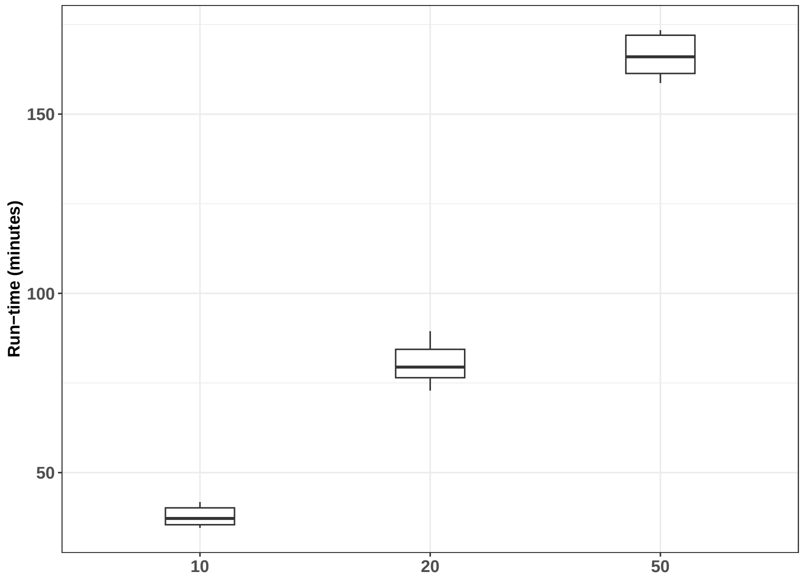

Figure 21: Runtime of time-dependent GDP for varying number of time points (nodes T). The truncation level of proposed GDP is fixed at $L = 10$. Boxplots show variation across 50 independent replicates.

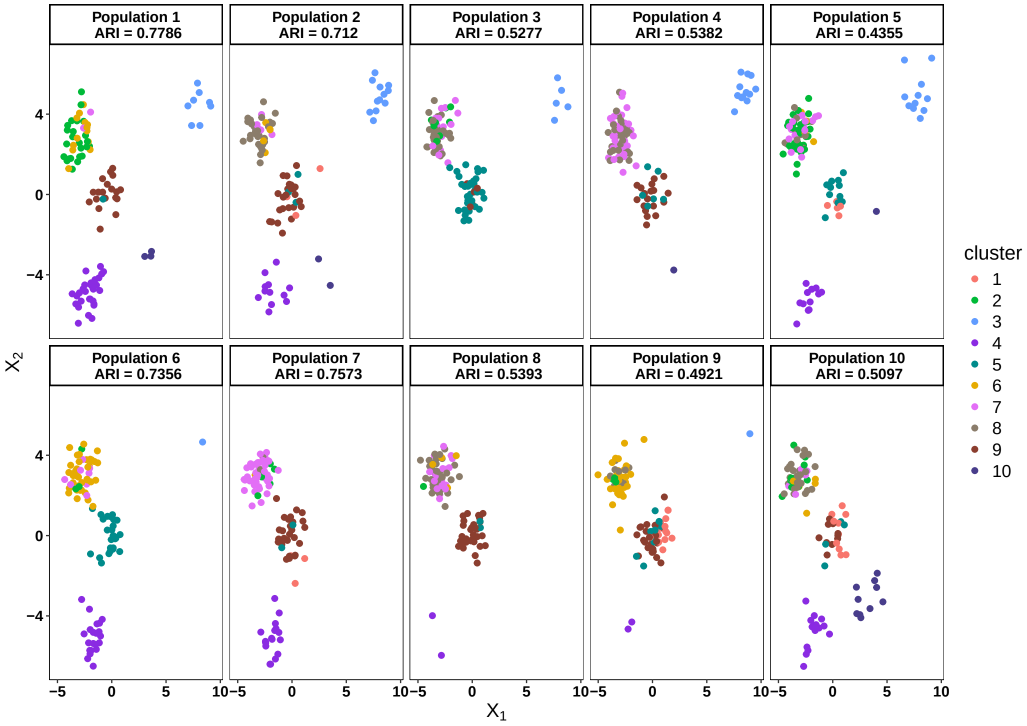

Figure 22: Clustering performance of time-dependent data using HDP for $T = 10$ time points. Population t refers to the observed group at time point t. The colors indicate the estimated clusters by HDP. Adjusted Rand index is reported at the top of each panel.

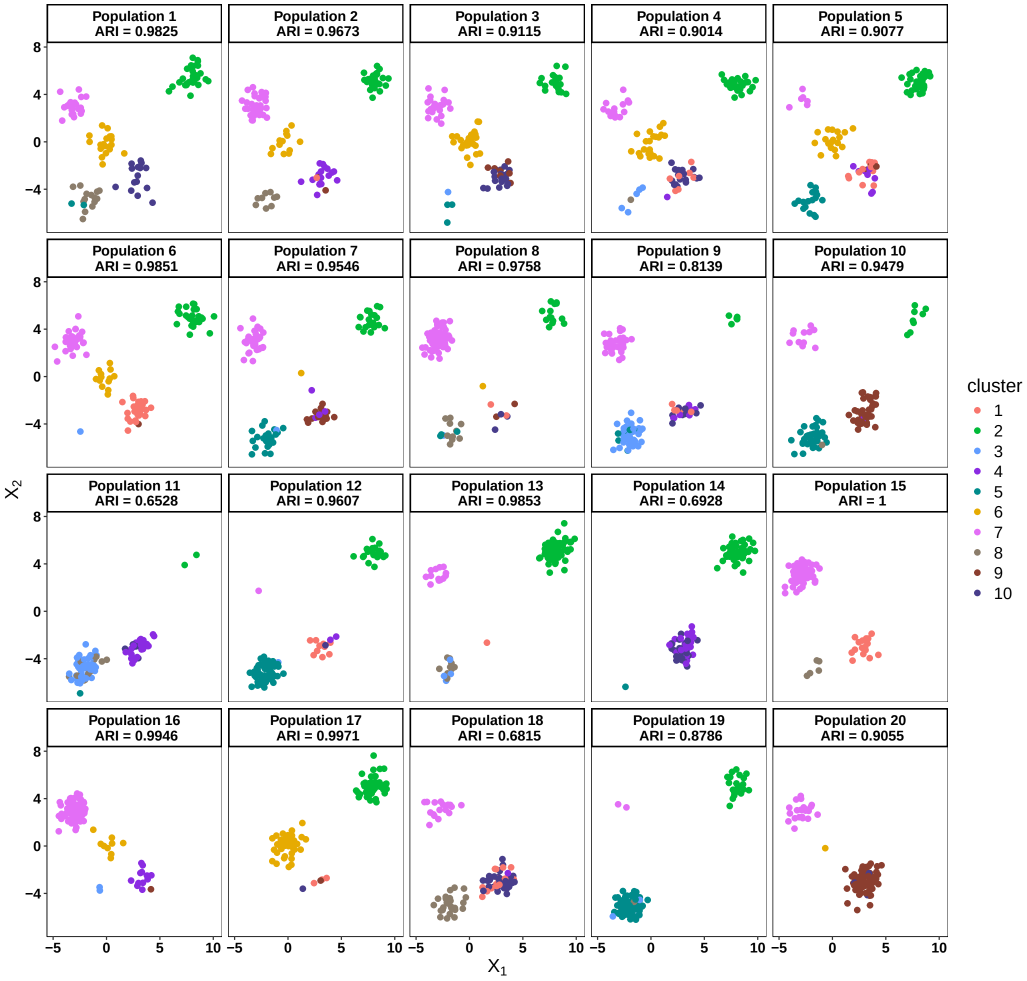

Figure 23: Clustering performance of time-dependent data using HDP for $T = 20$ time points. Population t refers to the observed group at time point t. The colors indicate the estimated clusters by HDP. Adjusted Rand index is reported at the top of each panel.

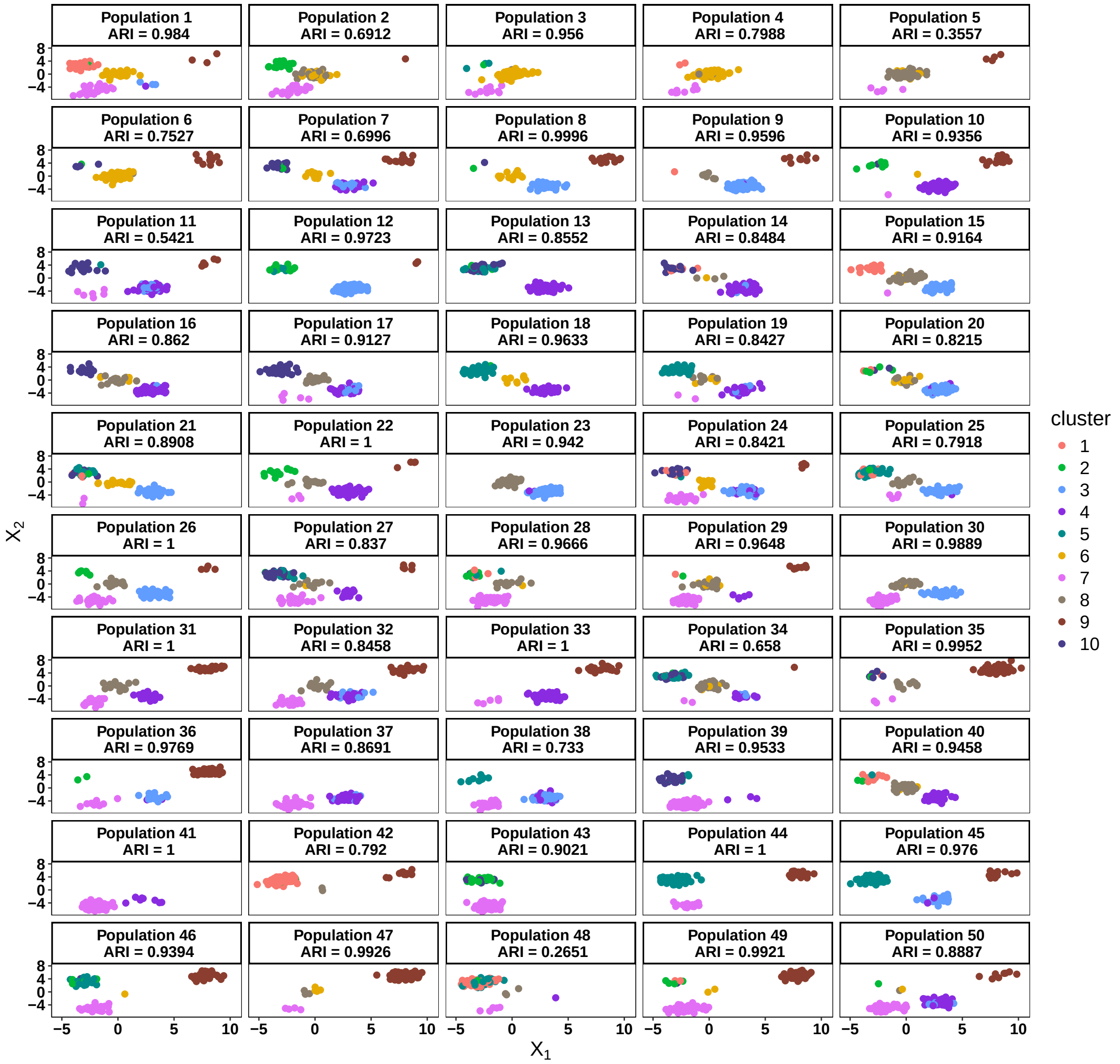

Figure 24: Clustering performance of time-dependent data using HDP for $T = 50$ time points. Population t refers to the observed group at time point t. The colors indicate the estimated clusters by HDP. Adjusted Rand index is reported at the top of each panel.

Appendix G. Real Data Analysis plots

Sensitivity. To study the effect of the truncation level of GDP, we varied $L = 10, 20, 30,$ and 50. We considered 50 independent replications and studied the estimated number of clusters for the different choices of the truncation level, L. The boxplots of the number of estimated clusters in Figure 25a shows that our method is relatively robust with respect to the truncation level, especially for $L = 30, 50$. Furthermore, Figure 25b shows that the runtime of our sampler is approximately linear in the truncation level of GDP. These results led us to consider the truncation level $L = 30$ for the real data analysis using GDP (the estimated number of clusters is well below 30), as reported in the main manuscript.

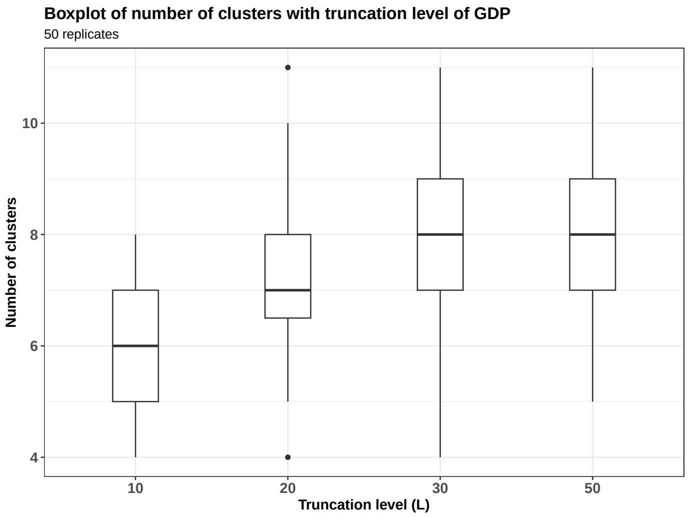

(a) Number of estimated clusters.

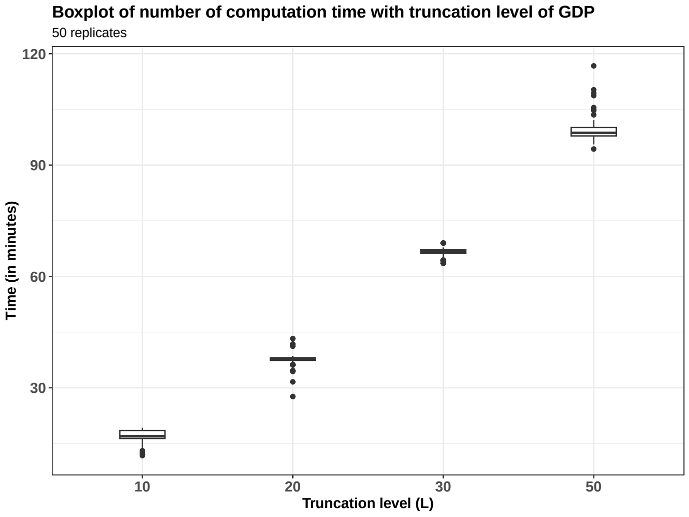

(b) Computational time.

Figure 25: Robustness and scalability of GDP for various choices of truncation level (L) for the real data over 50 independent replications.

For our real data analysis, we ran four parallel chains of the Gibbs sampler for $50,000$ iterations. The traceplots (Figure 26) of the log-likelihood for each of the four parallel chains of our sampler, after discarding the initial $35,000$ samples and thinning the samples by a factor of 15 indicated no lack of convergence of our sampler. Furthermore, the traceplots indicate the presence of local modes, necessitating the need to concatenate posterior samples across these chains for more efficient and reliable inference.

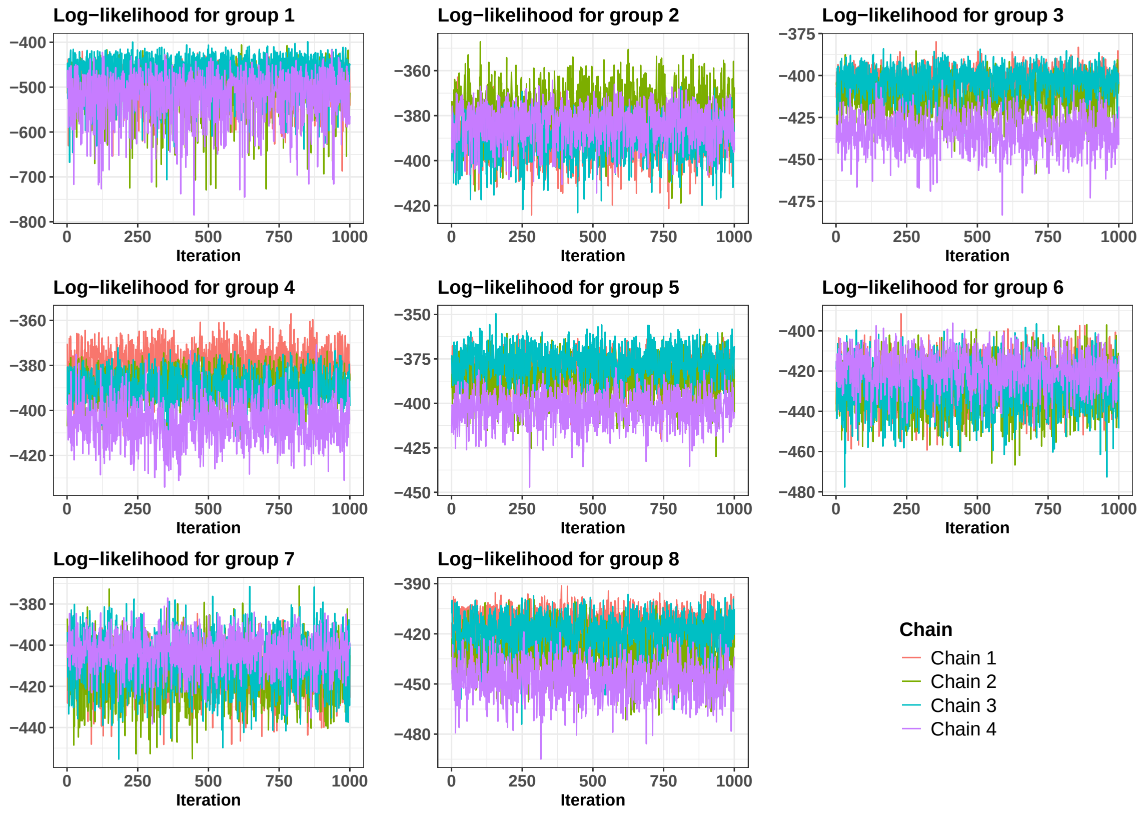

(a) GDP

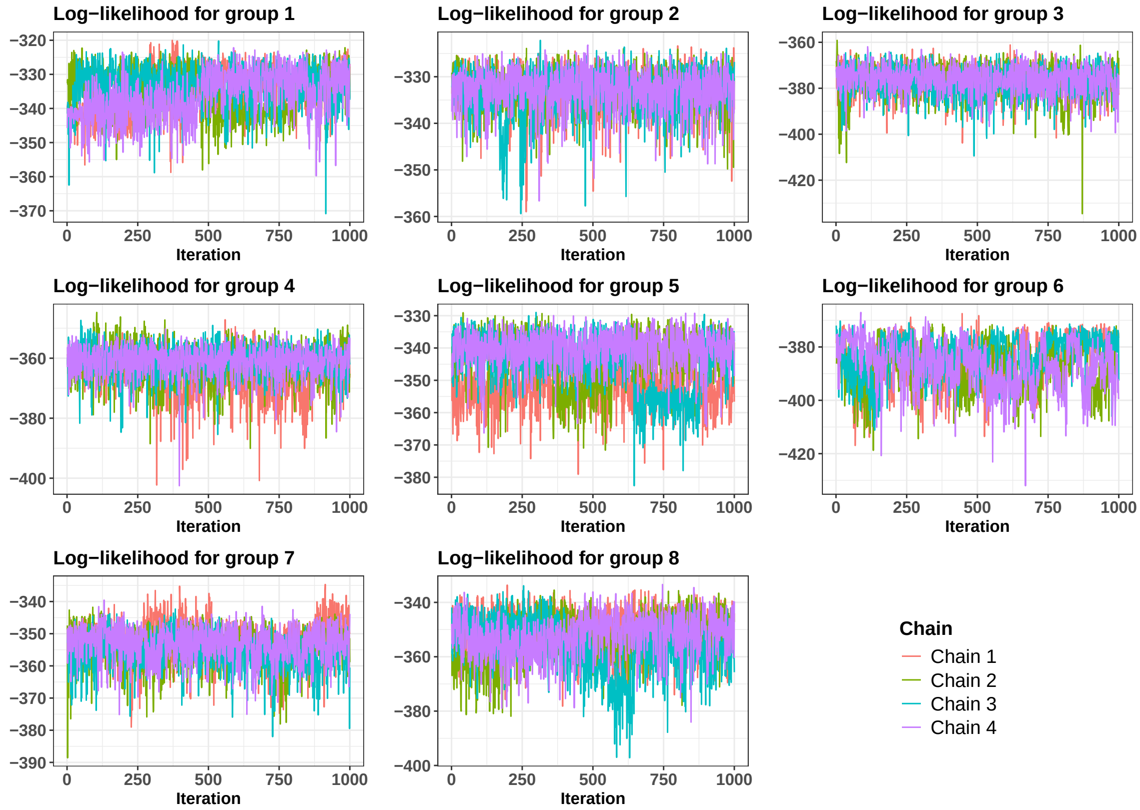

(b) HDP

Figure 26: Group-specific traceplots of log-likelihood for four parallel chains of our MCMC for (a) GDP and (b) HDP.
